# Generation of pulsatile ERK activity in mouse embryonic stem cells is regulated by Raf activity

**DOI:** 10.1038/s41598-023-36424-6

**Published:** 2023-06-10

**Authors:** Yayoi Toyooka, Kazuhiro Aoki, Fumiko Matsukawa Usami, Sanae Oka, Azusa Kato, Toshihiko Fujimori

**Affiliations:** 1 Division of Embryology, National Institute for Basic Biology, National Institutes of Natural Sciences, 5-1 Higashiyama, Myodaiji-Cho, Okazaki, Aichi 444-8787 Japan; 2 Department of Clinical Application, Center for iPS Cell Research and Application (CiRA), Kyoto University, 53 Kawahara-Cho, Shogoin, Sakyo-Ku, Kyoto 606-8507 Japan; 3 Division of Quantitative Biology, National Institute for Basic Biology, Okazaki, Japan; 4 Quantitative Biology Research Group, Exploratory Research Center on Life and Living Systems (ExCELLS), Okazaki, Aichi Japan; 5 Department of Basic Biology, School of Life Science, SOKENDAI (The Graduate University for Advanced Studies), Okazaki, Aichi Japan

**Keywords:** Stem cells, Embryonic stem cells, Pluripotent stem cells, Stem-cell differentiation

## Abstract

The extracellular signal-regulated kinase (ERK) is a serine/threonine kinase that is known to regulate cellular events such as cell proliferation and differentiation. The ERK signaling pathway is activated by fibroblast growth factors, and is considered to be indispensable for the differentiation of primitive endoderm cells, not only in mouse preimplantation embryos, but also in embryonic stem cell (ESC) culture. To monitor ERK activity in living undifferentiated and differentiating ESCs, we established EKAREV-NLS-EB5 ESC lines that stably express EKAREV-NLS, a biosensor based on the principle of fluorescence resonance energy transfer. Using EKAREV-NLS-EB5, we found that ERK activity exhibited pulsatile dynamics. ESCs were classified into two groups: active cells showing high-frequency ERK pulses, and inactive cells demonstrating no detectable ERK pulses during live imaging. Pharmacological inhibition of major components in the ERK signaling pathway revealed that Raf plays an important role in determining the pattern of ERK pulses.

## Introduction

Mouse embryonic stem cells (ESCs) are pluripotent cell populations established from the preimplantation embryo^1,2^, and have been used to study the in vitro differentiation of many cell types in the developing embryo. ESCs have also been useful in research on the cell lineages of extraembryonic tissues such as the trophectoderm and primitive endoderm (PrE), both of which arise in preimplantation stages and finally form the placenta and the extraembryonic membranes that are essential for fetal nutrition and gas exchange^3,4^. PrE mainly contributes to extraembryonic membranes such as the amnion. ESCs differentiate rapidly and directly into PrE cells, with no intermediate cell types, and have been used as an in vitro model of PrE differentiation in the inner cell mass (ICM) of preimplantation embryos.

Extracellular signal-regulated kinase (ERK) is a serine/threonine kinase that is known to regulate a wide range of cellular events, such as cell proliferation and differentiation, in response to growth factors and differentiation factors^5^. The Ras-Raf-MEK-ERK signaling pathway is thought to be indispensable for PrE differentiation in preimplantation embryos. Pharmacological inactivation of the ERK pathway in mouse preimplantation embryos blocks PrE differentiation and directs ICM cells toward epiblast (EPI) cells, which are a pluripotent cell population, giving rise to the embryo proper^6,7^. Further, mouse embryos in which ERK signaling pathway genes were inactivated exhibited defects in the development of PrE or its derivatives^8,9^. Since embryos lacking Fgf4^10,11^ and Fgfr1 and Fgfr2^12,13^ show defects in the development of PrE lineages as well as peri-implantation lethality, Fgf4, Fgfr1, and Fgfr2 are thought to lie upstream of the ERK signaling pathway in the preimplantation embryo. Fgf4, which is secreted from ICM cells in an autocrine fashion, binds to Fgfr1 and Fgfr2 and activates ERK. The disruption of either Fgfr1 or Fgfr2 alone does not cause defects in the formation of PrE cells, while double knockout of Fgfr1 and Fgfr2 prevents PrE differentiation at the late blastocyst stage^12,13^. These results suggest that Fgfr1 and Fgfr2 play redundant roles in PrE determination. Fgf4-Fgfr-mediated ERK activation is believed to play a critical role in causing individual ICM cells to exclusively express Nanog and Gata6, which are transcription factors directing differentiation toward EPI or PrE cells, respectively^3^.

ERK activity is also indispensable for in vitro mouse ESC differentiation, including PrE cell differentiation. Pharmacological inhibition of Fgf-ERK signaling blocks ESC differentiation^6^, and Fgf4 knockout ESCs were shown to fail to differentiate^14,15^. In addition, ESCs lacking Grb2, a component of the ERK signaling pathway, fail to differentiate into PrE cells^16^. Moreover, activation of the ERK pathway by overexpression of a constitutively active form of Ras causes ESCs to differentiate into PrE cells^17,18^, suggesting that activation of the ERK pathway is sufficient for inducing PrE cell differentiation.

In this report, we visualized and quantitatively analyzed ERK activity in ESCs by live-cell imaging with a biosensor based on the principle of fluorescence resonance energy transfer (FRET). We identified two types of cell populations in undifferentiated ESC cultures: those in which ERK signaling was activated and those in which it was not. We observed pulsatile ERK activity in active cells, and the patterns of the pulses were disrupted by the addition of a Raf inhibitor, suggesting an important role of Raf signaling in modulating ERK activity in pluripotent ESCs.

## Results

### Pulsatile ERK activity in undifferentiated ESCs was observed using a FRET biosensor

**Figure 1:**
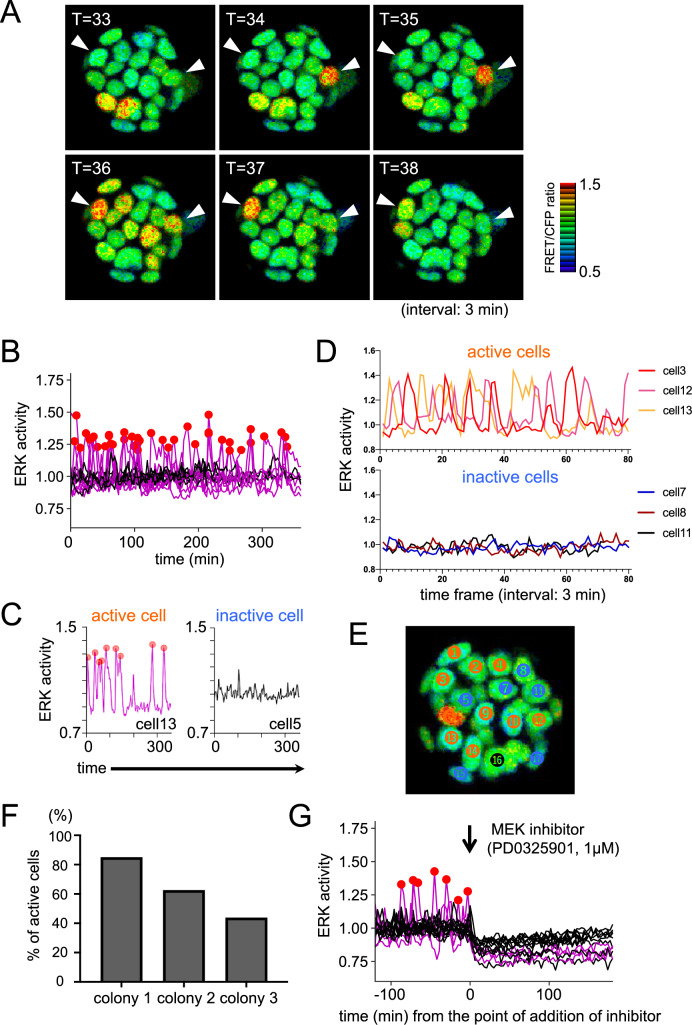
(**A**) FRET/CFP images of live-imaging of a undifferentiated EKAREV-NLS-EB5 ESC colony. Arrowheads indicate nuclei of cells showing high FRET/CFP ratio during time frame for data acquisition (T) = 33–38 (99–114 min)). Interval of each T is 3 min. (**B**–**E**) Analysis results of the same colony as shown in A. (**B**) Result of peak detection analysis by python as a representative example of the 8 analyzed colonies. Vertical axis indicates ERK activity (FRET (YFP)/CFP ratio). (**C**) Examples of cells classified as an ERK-active (left, showed in pink lines) and an inactive (right, black lines) cells. Red circles indicate detected peaks. (**D**) Raw data of FRET/CFP ratio of three typical examples for ERK-active (upper) and inactive (bottom) cells in the colony. (**E**) Distribution of active/inactive cells in the colony. Cells classified as active cells are indicated in red, and inactive cells are indicated in blue, respectively (N = 16). The cell divided during live imaging (in this case, cell 16) was excluded from analysis because signals of CFP and YFP fluorescence in dividing cells were decreased and assumed not to measure accurate ERK activity. (**F**) Percentage of active cells in representative three colonies in maintenance (+ LIF) condition. Ratios of active/inactive cells in colony 1, 2, and 3 are 17/3 (N = 20), 10/6 (N = 16), and 7/9 (N = 16), respectively. (**G**) Result of peak detection analysis of data of FRET/CFP ratio before and after addition of MEK inhibitor PD0325901 (1 μM) in maintenance (+ LIF) condition. Arrow indicates the time point of addition of inhibitor (between T = 40 and 41 (120–123 min).

To monitor ERK signaling activity in individual ESCs, we used the Piggybac system to introduce the FRET biosensor EKAREV-NLS^19,20^ into the mouse ESC line EB5^21^ (kindly provided by Dr. Niwa). In this cell line, one of the Oct4 loci is replaced with a blasticidin resistance gene, and therefore differentiating (Oct4-negative) cells can be excluded in the presence of blasticidin S. We confirmed that cloned EKAREV-NLS-EB5 ESC lines stably expressing EKAREV-NLS exhibited a sufficient expression level of the FRET biosensor (Fig. S1A). First we performed live imaging of EKAREV-NLS-EB5 ESCs under maintenance conditions (serum + LIF), using blasticidin S to eliminate differentiating cells from the culture. We acquired live imaging data of CFP and FRET (YFP) fluorescence, and calculated the ratio of FRET (YFP)/CFP intensities as a proxy for ERK activity (Fig. S1B). The value of FRET (YFP)/CFP ratio in the nucleus of each cell at each time frame of live imaging was measured. We observed pulsatile ERK activation with a pulse width of about 10 min (Fig. 1A,B,D and supplementary movie 1), as previously seen in several somatic cell lines^19^.

### Heterogeneity of ERK signaling activity in undifferentiated ESC colonies

We observed several undifferentiated ESC colonies and found that cells could be classified into two populations: those that did or did not demonstrate obvious ERK activation pulses during live imaging. We defined time points with a FRET/CFP ratio above 1.2 as peaks, and performed peak detection of each cell by python (Fig. 1B). We found that some cells exhibit no obvious peaks even they were in the same colony which contained cells showing obvious peaks (Fig. 1C and D). Then we analyzed number of peaks (pulses) in each cells that we could continuously measure FRET/CFP ratio for more than 120 min. We defined cells exhibiting one or more pulses during live imaging as “active cells,” and those showing no obvious pulses throughout live imaging as “inactive cells” (Fig. 1C and D). Active and inactive cells seemed to be randomly distributed in the colony (Fig. 1E). The number of active/inactive cells in the colony used for analysis shown in Fig. 1A–E (colony 2) was 10/6 (63% was active cells, Fig. S1C), but the ratio was varied between colonies (Fig. 1F). We analyzed 8 colonies cultured in maintenance condition in total including data of additional experiments (Fig. S2), and found that 89 out of 161 cells (55.3%) were active cells. A histogram of inter-pulse intervals demonstrated an exponential distribution (Fig. S1D), suggesting that pulse generation in active cells occurred in a stochastic manner. The ERK activation pulses observed in undifferentiated ESCs completely disappeared in the presence of MEK inhibitor PD0325901 (Fig. 1G, S1E, and supplementary movie 2), proving that these pulses accurately reflected ERK activity. We notice that a concomitant decrease in the baseline of all cells was observed, it is assumed that the activation level of ERK in inactive cells is also MEK-dependent (Fig. 1G and S1E). Because activation of ERK signaling results in PrE differentiation of ESCs^17,18^, we speculated that the ERK-active population constituted a subpopulation that would be close to exit from pluripotency and ready to initiate differentiation, while inactive cells were a fully pluripotent, grand state subpopulation of ESCs.

### Some cells within the same undifferentiated colonies exhibit synchronized ERK pulse patterns

**Figure 2:**
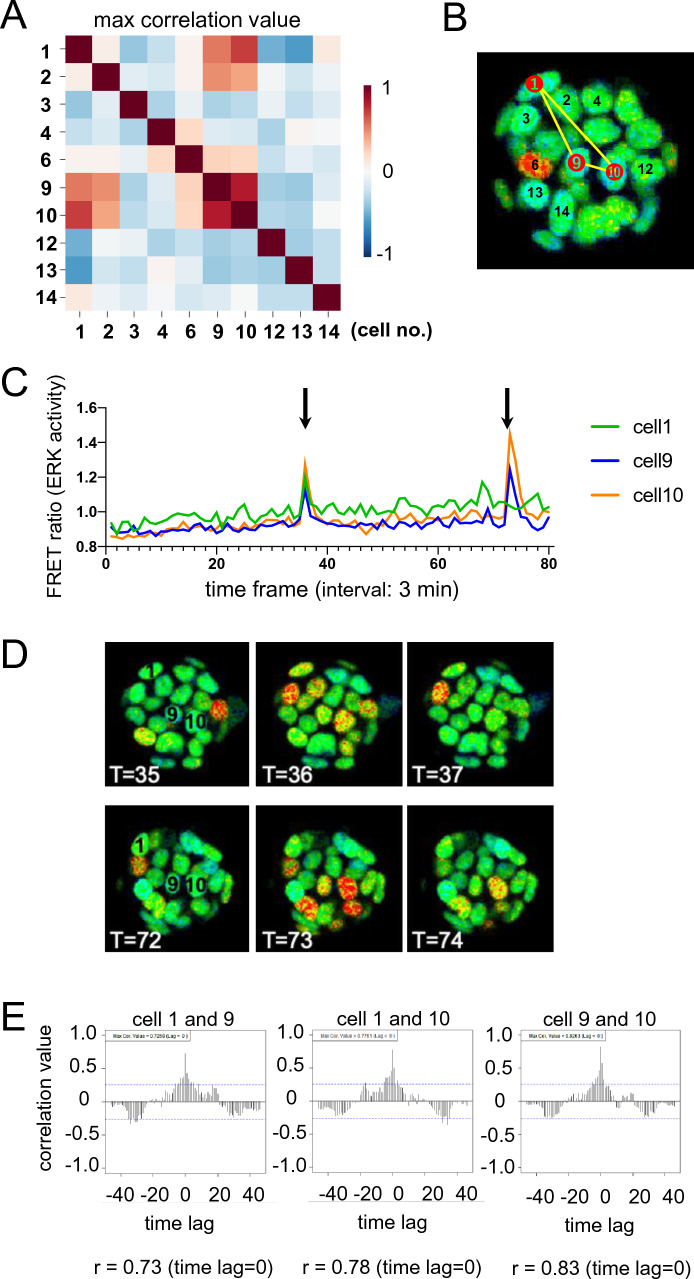
An example for correlation of peak generation patterns among active cells in the colony. Result of one of eight analyzed colonies is shown as a representative example. (**A**) Result of cross-correlation analysis of all active cells in the colony. Data from the first 120 min was used for analysis. Note that strong correlation was observed between cell 1, 9, and 10. (**B**) Numbers of cells only classified as active cells in the colony. Numbers of cells that showed high correlation are indicated in red, and connected by yellow lines. (**C**) Raw data of FRET ratio of cell 1, 9, and 10. Cell 1, 9, and 10 synchronously generated pulses at T = 35–37 (105–111 min, left arrowhead), and cell 9 and 10 synchronously generated pulses at T = 72–74 (216–222 min, right arrowhead). (**D**) Actual FRET/CFP images of the colony at T = 35–37 and at T = 72–74. (**E**) Results of cross-correlation analysis between cell 9 and cell 1, and cell 9 and cell 10. Note that especially strong correlation was observed between pulse patterns of cell 9—cell 1 (r = 0.73) and cell 9—cell 10 (r = 0.83) without time lag. Vertical axis indicates cross-correlation, horizontal axis indicates time lag.

Through the observation of ERK activity in undifferentiated ESCs, we found that several cells in the same colony sometimes showed synchronized pulse patterns. In the case of the colony shown in Fig. 2, which is the same as shown in Fig. 1, it was found that pulse pattern of cell 1, 9, and 10 showed relatively strong correlation by correlation analysis (r > 0.7, Fig. 2A and E). Cell 9 and 10 were adjacent, while cell 1 was not neighboring to 9 and 10 during live imaging (Fig. 2B and supplementary movie 1). Cell 1, 9, and 10 at around time frame for data acquisition (T) = 35–37 (105–111 min), and cell 9 and 10 at around T = 72–74 (216–222 min) could be seen to emit obvious synchronized ERK pulse (Fig. 2C and D). Interestingly, pulse patterns of these cells were found to synchronized with no time lag (Fig. 2E) although cell 1 was in a relatively distant position and was separated from cell 9 and 10 by other cells (Fig. 2B). We analyzed several other colonies and also found similar pair or groups of cells showing synchronized pulse patterns within time lag of + 25 to − 25 min (Fig. S3). Cross-correlation analysis was performed on data from a total of 161 cells (8 colonies), and it was found that the ERK activity pulse patterns of 108 cell pairs showed correlation with cells in the same colony. We hypothesized that cell pairs or groups exhibiting synchronized pulse patterns may have originally divided from the same cell, and measured and analyzed the ERK activity of cells that occasionally underwent cell division during live imaging. However, daughter cells did not subsequently exhibit synchronized pulse patterns (data not shown), there is likely no relation between cell division and synchronized pulse patterns.

### ERK activity in ESCs exposed to excess FGF4 in serum-containing cultures

**Figure 3:**
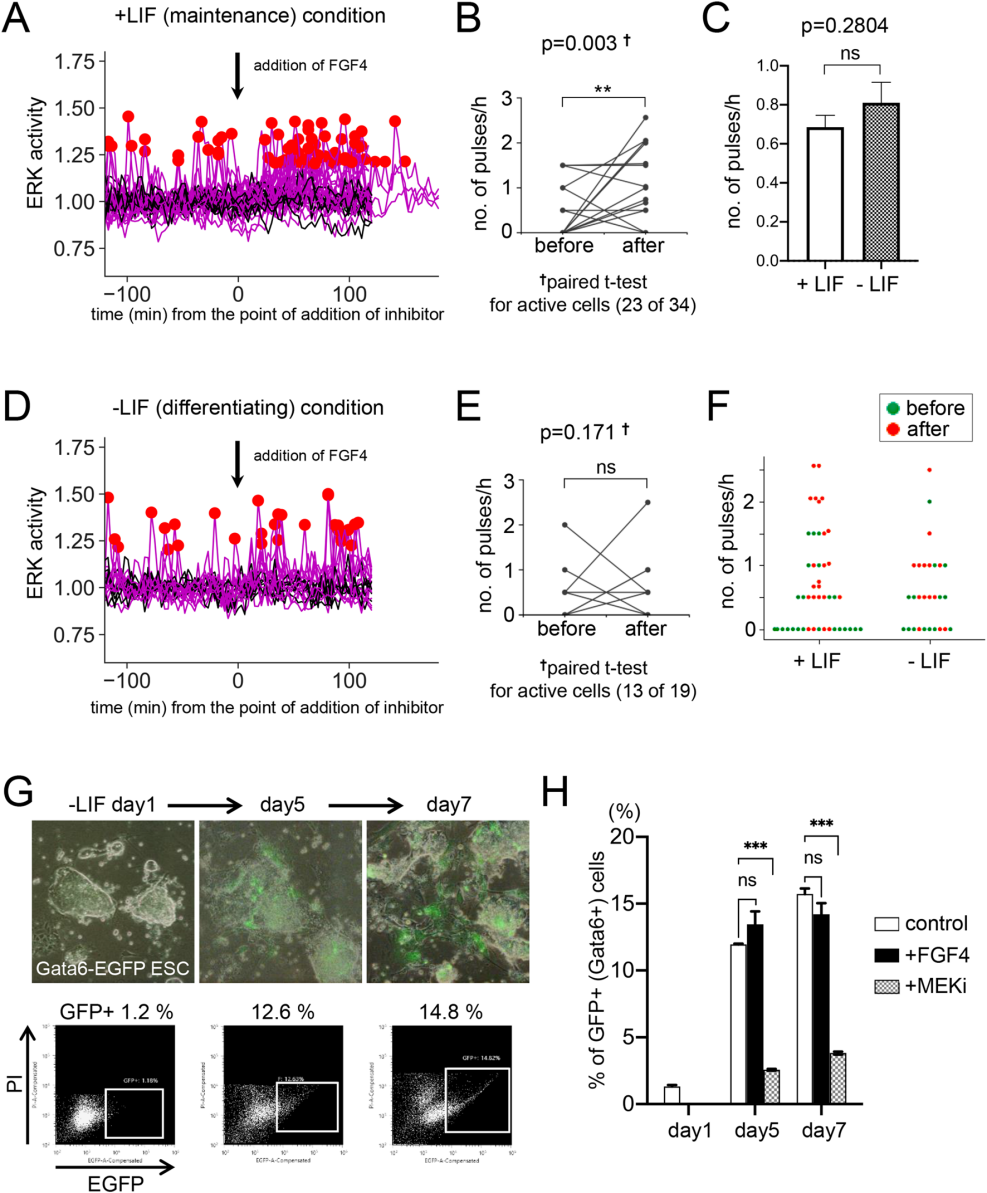
(**A**) Result of peak detection analysis of FRET/CFP data in maintenance (+ LIF) condition added excess amount of (500 ng/ml) recombinant FGF4 and 1 μg/ml heparin at the time point between T = 40 and 41 (120–123 min) (N = 34, from more than 3 colonies). (**B**) Before-after graph for ERK activity in each active cell before and after addition of FGF4 in + LIF condition. Vertical axis indicates number of pulses/hour. Note that only results of active cells (showing more than one peak during 240 min live-imaging, 23 of 34 cells) are plotted. Significant differences between before and after were determined by t-test. p value of paired t-test (*P* = 0.003) is shown in the graph. (**C**) Comparison of frequency of ERK pulse under + LIF (N = 89, from 8 colonies) and -LIF (N = 37, from 11 colonies) conditions. Data of the first 120 min of active cells in both conditions were used for analysis. Vertical axis indicates number of pulses/hour. Means of pulses/hour in + LIF and -LIF conditions are 0.69 and 0.81, respectively. Result of t-test (*P* = 0.2804) is presented in the graph. (**D**) Result of peak detection analysis of FRET/CFP data in differentiating condition (1 day after withdrawal of LIF) added FGF4 and heparin at the time point between T = 40 and 41 (120–123 min) (N = 19, from more than 3 colonies). (**E**) Before-after graph for ERK activity in each active cell before and after addition of FGF4 in -LIF condition. Only results of active cells (13 of 19 cells) are plotted. Result of paired t-test (*P* = 0.171) for before and after addition of FGF4 is shown in the graph. (**F**) Swarm plot showing frequency of ERK pulse of individual active cell before (green dots) and after (red dots) addition of FGF4 in + LIF (N = 23) and -LIF (N = 13) conditions. Vertical axis indicates number of pulses/hour. (**G**) An example for analysis of Gata6 + PrE cells differentiated from a Gata6-EGFP reporter ESC line in the presence of excess amount of (500 ng/ml) FGF4 and heparin in -LIF condition. PI, Propidium Iodide. (**H**) Result of FACS analysis of Gata6 + PrE cells differentiated from a Gata6-EGFP reporter ESCs. Means and standard errors from 3 experiments are shown. One-way ANOVA with Dunnett's multiple comparisons test was performed to verify statistical significant differences. MEKi, MEK inhibitor PD0325901 (1 μM). Significance levels of statistical tests are indicated as *P < 0.05; **P < 0.01; ***P < 0.001. ns indicates not significant (*P* > 0.05).

The local concentration of Fgf4 in ICM is thought to be critical for PrE determination in mouse preimplantation embryos. Administration of an excess concentration of recombinant FGF4 (500 ng/ml) with heparin resulted in an increased proportion of PrE cells among ICM cells^7^, and Fgf4 ± embryos possessed more EPI cells than wild-type embryos^22^. We examined whether addition of an excess amount of recombinant FGF4 to ESC culture in maintenance (+ LIF) or differentiating (-LIF) conditions during live imaging increased ERK pulses in active cells, and also whether the number of ERK-active cells was increased and then altered the differentiation efficiency into the PrE cell lineage in -LIF condition. Before starting the experiment, we confirmed that EB5 cells in the differentiating condition could differentiate into Gata4-positive PrE cells within 3 days (Fig. S4). First, we performed live imaging of ERK activity in + LIF or -LIF condition for 240 min, and added an excess concentration of recombinant FGF4 (500 ng /mL) to the culture at the point T = 40–41 (at 120 min from start) of live imaging. In + LIF condition, the frequency of ERK activation pulses was significantly increased after addition of excess FGF4 (Fig. 3A,B,F and supplementary movie 3, *P* = 0.003 by paired t-test for before-after addition of FGF4). The ratio of ERK-active/inactive cells classified from the results of all time frames of live imaging was 23/9 (72% was active cells), not much different from those of colonies in maintenance condition (Fig. 1F), it seemed that excess FGF4 did not drastically affect ratio of active/inactive cells. Next, we examined the effect of LIF withdrawal on the pattern of ERK activation pulses and the active/inactive state of the cells. We performed live imaging of cells in -LIF condition (1 day after removal of LIF), calculated and compared the frequency of ERK pulses (number of pulses/hour) in active cells in differentiating culture and maintenance (+ LIF) culture. We found that the ratio of active/inactive cells was not significantly different in both culture conditions (55.3% active in + LIF, the ratio of active/inactive cells was 89/72 (from 8 colonies), 43.5% active in -LIF, the ratio of active/inactive cells was 37/48 (from 11 colonies), Fig. S1C, S3A, S2, and S5), and also that the frequency of ERK pulses did not significantly differ (Fig. 3C, *P* = 0.2804), suggesting that withdrawal of LIF had considerable effect neither on the frequency of ERK pulses nor active/inactive status.

Contrary to the result of + LIF condition, the frequency of ERK pulses in the -LIF differentiating condition after addition of excess FGF4 was not markedly different (Fig. 3D,E, and F, *P* = 0.171 by paired t-test for before-after addition of FGF4), and the percentage of active/inactive cells also have not affected (13/19, 68% active). Finally, we examined whether excess FGF4 affects the differentiation efficiency of ESCs into PrE cells using Gata6-EGFP reporter ESCs. Gata6-EGFP reporter ESCs were derived from a Gata6-EGFP reporter mouse line that was confirmed to exhibit GFP fluorescence in tissues where Gata6 expression has been reported^23,24^, such as the PrE layer of preimplantation embryos, PrE derivatives in post-implantation embryos, and the intestine of adult mice (Fig. S6A–D). Expression of EGFP in the PrE layer of embryoid bodies derived from Gata6-EGFP reporter ESCs was also confirmed (Fig. S6E). From the results under the -LIF differentiating condition, we found that the presence of excess FGF4 (500 ng /mL) did not significantly alter the efficiency of Gata6 + PrE differentiation compared to those of control (Fig. 3G and H). Addition of MEK inhibitor PD0325901 to the culture significantly decreased the efficiency of PrE differentiation, indicating that activation of ERK is essential for PrE differentiation of this ESC line. These data suggest that excess FGF4 did not affect immediate ERK activity, the active/inactive cell state, or the differentiation efficiency of PrE of ESCs in our -LIF differentiating culture conditions.

### Raf activity determines the ERK pulse width and may modulate the active/inactive state

**Figure 4:**
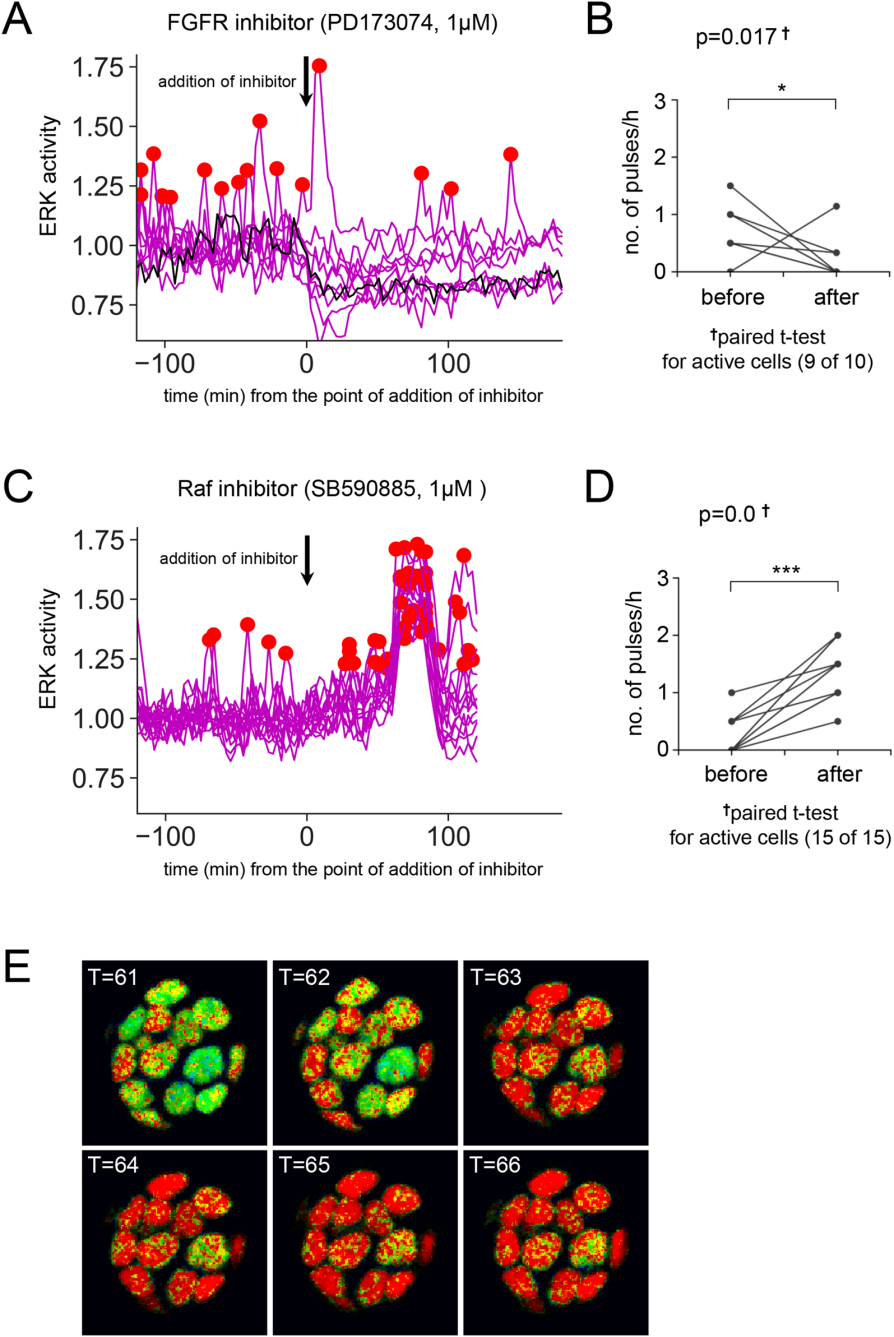
(**A**) Result of peak detection analysis of FRET data in maintenance (+ LIF) condition before and after addition of FGFR inhibitor PD173074 (1 μM) at the time point between T = 40 and 41 (120–123 min) (N = 10). (**B**) Before-after graph for ERK activity in each active cell before and after addition of FGFR inhibitor in + LIF condition. Vertical axis indicates number of pulses/hour. Note that only active cells (9 of 10 cells) are plotted. Result of paired t-test (*P* = 0.017) is shown in the graph. (**C**) Result of peak detection analysis of FRET data in + LIF condition before and after addition of Raf inhibitor SB590885 (1 μM) at the time point between T = 40 and 41 (120–123 min) (N = 15). Data from 2 out of 9 analyzed colonies is shown. (**D**) Before-after graph for ERK activity in each active cell before and after addition of Raf inhibitor in + LIF condition. Only active cells (15 of 15, in this case, all cells) are plotted. Result of paired t-test (*P* = 0.0) is shown in the graph. (**E**) FRET ratio images after addition of Raf inhibitor SB590885 (T = 61–66 (183–198 min)) in + LIF condition. Significance levels of statistical tests are indicated as *P < 0.05; **P < 0.01; ***P < 0.001.

To explore the factor(s) regulating the generation of ERK pulses and the active/inactive state in undifferentiated ESCs, we examined the effect of inhibiting molecules that located upstream of the ERK pathway and regulating ERK activity. We performed live imaging of ERK activity in the maintenance condition, then added inhibitors to the culture medium 120 min after we began acquiring FRET/CFP images (at T = 40–41). First, we added FGFR inhibitor PD173074 (1 μM), and observed that the FGFR inhibitor suppressed the generation of ERK pulses in active cells which exhibiting pluses in the first half of live imaging (Fig. 4A, B, and S9, *P* = 0.017 by t-test for before-after addition). This result suggests that activation of FGFRs is indispensable for generating ERK activation pulses in undifferentiated ESCs. In somatic cells, ERK signaling pathway is known to be activated by EGFR and to promote cell proliferation^19^, but we found that an EGFR inhibitor had almost no effect on ERK activity in ESCs (Fig. S7A and B). Finally, we added Raf inhibitor SB590885 (1 μM), and found that about 60 min after addition of Raf inhibitor, a wide pulse (18–20 min) was simultaneously observed in all cells (Fig. 4C,D,E, S7E, S8, S9, and supplementary movie 4). The shape of the ERK pulse, especially its width, was drastically affected, and significantly wide pulses were observed in all cells including cells exhibiting no pulse in the first half of live imaging (Fig. 4C,D, S7E, and S8). The widened pulse was recognized as an aggregate of pulses by peak detection analysis (Fig. 4C,D, S7E, and S8). Such a widened, synchronized pulse was never observed in case that DMSO (solvent for all inhibitors), EGFR inhibitor, or FGFR inhibitor was added (Fig. 4A, S7A, and C). Since Raf inhibitors are known to activate Raf (a phenomenon called “paradoxical activation”^25^), and given the mechanism of action of Raf in the MAPK signaling pathway, we speculate that the generation of the wide pulse triggered by Raf inhibitor SB590885 is the result of Raf activation. This result indicates that Raf activity modulates the pattern of ERK pulses in both of active and inactive cells.

## Discussion

In this study, we established mouse ESC lines expressing the FRET biosensor EKAREV-NLS, and measured ERK signaling activity in individual ESCs in maintenance and differentiating conditions. In our culture system, ERK activity was detected as pulsatile FRET/CFP signals. We found that undifferentiated ESCs could be classified into two populations, characterized by the presence or absence of FRET/CFP signal pulses during live imaging (Fig. 1A–E, and supplementary movie 1). We defined these two populations as “active cells” and “inactive cells,” respectively.

Previous studies that monitored ERK activity in mouse ESCs were performed by Deathridge et al. and Raina et al.^26,27^. As in our study, Deathridge et al. observed the ERK activity of individual ESCs using the FRET biosensor EKAREV-NLS, but they detected FRET signals not as pulses, but as gentle increments and decrements of the FRET level^26^. They detected fluorescence signals with an inverted microscope connected with EMCCD camera because the probe signals were too weak to be identified with a confocal microscope^26^. This indicates that the expression level of the probe in cells may have been too low to detect FRET pulses due to the gene transfer conditions that were used. However, the authors also reported that ESCs exhibited heterogeneous ERK activity, suggesting varying FRET signal values. On the other hand, Raina et al. utilized an ERK-KTR-mClover probe that could detect ERK activity as the ratio of cytoplasmic to nuclear mClover fluorescence, and identified ERK activity as a series of short pulses similar to those observed in our study^27^.

It has been reported that several genes, such as *Nanog* and *Rex1*, are expressed heterogeneously in pluripotent ESCs, with high expression indicating an increased propensity for self-renewal and low expression reflecting a tendency toward differentiation^28–30^. Nanog is a transcription factor that is critical for the maintenance of pluripotency^31,32^, and it is heterogeneously expressed in mouse ESCs^28^. Rex1 is also a transcription factor that is heterogeneously expressed in ESC cultures^30^, but it is not thought to be essential for maintaining pluripotency^33^. High and low Nanog states are reversible in maintenance culture containing serum, and low-Nanog ESCs are closer to differentiation than high-Nanog ESCs^28^. We assumed that the ERK-active and -inactive cells observed in our system corresponded to low- and high-Nanog cells, respectively, since active cells were considered to be close to differentiation based on the fact that activation of the ERK pathway in ESCs by the constitutively active form of Ras results in differentiation to the PrE lineage^17,18^, and also because expression of Nanog is suppressed by the ERK signaling pathway^6,7,9^.

We tried to purify ERK-active and -inactive cells using fluorescence-activated cell sorting (FACS) as those with a high or low FRET/CFP ratio and to compare Nanog expression levels between those populations, but failed to detect difference between two populations. (data not shown). Deathridge et al. used Nanog-EGFP and Rex1-EGFP reporter ESC lines to examine the association between ERK activity and high or low Nanog or Rex1 expression, but they found no significant correlation^26^. Thus, switching between the ERK-active and -inactive states might not be immediately associated with the expression level of Nanog or Rex1.

In our system, active and inactive cells seemed to be randomly distributed (Figs. 1E, 2B and S3B). Deathridge et al. also examined whether local differences in cell signaling explained the presence of active and inactive ESC populations, and concluded that distinct ERK activity dynamics were randomly and spatially distributed in the ESC colony^26^ Interestingly, we found that several pairs or groups of active cells in the same colony showed significantly synchronized ERK pulse patterns (Fig. 2A–E and S3A–E). Active cells with synchronized pulses were not always adjacent to each other, implying that a signal from some diffusible factor might cause this synchronization. It may be possible that there are gradation of Fgf4 concentration in the colony, and only active cells that catch a wave of Fgf4 in concentration above a certain threshold generate ERK pulses simultaneously.

Raina et al. established Fgf4−/− ESC line harboring sensor for ERK activity to control FGF4 concentration externally, and showed that the addition of FGF4 increased the percentage of pulsing (active) cells in a dose-dependent manner using Fgf4−/− ESC line harboring sensor for ERK activity^27^. This result can be interpreted as that the concentration of FGF4 affects active/inactive status, but it also suggests that some cells generate ERK pulses and some are not responsive even when they are exposed to exactly the same concentration of FGF4 because ratio of pulsing and non-pulsing cells did not changed in an all-or-none fashion in their experimental system^27^. From that result, it is possible that other factors determine reactivity of each cell to FGF4, namely, active/inactive state.

We also examined if the addition of excess FGF4 to ESC culture in maintenance (+ LIF) and differentiating (-LIF) conditions altered the patterns of ERK pulse activity, ratio of active/inactive cells in the colony, and affected the differentiation efficiency into PrE cells. Excess FGF4 significantly changed frequency of ERK pulse in + LIF condition (Fig. 3A,B, and F). A previous study found that adding an excess amount of recombinant FGF4 (500 ng/ml) resulted in an increase in the proportion of PrE cells among ICM cells^7^. In our system, ERK pulse activity was affected by the addition of the same amount of recombinant FGF4 in + LIF condition (Fig. 3A,B, and F). However, frequency of ERK pulse activity was not increased by the addition of FGF4 in -LIF condition (Fig. 3D,E, and F), and the efficiency of differentiation of ESCs to the PrE cell lineage was also not significantly affected by large amounts of FGF4 (Fig. 3H). As described above, the local amount of Fgf4 in the ICM is thought to critically impact the fate of PrE/EPI cells in mouse embryos. Like ICM cells, ESCs secrete Fgf4 in an autocrine manner in preimplantation embryos, and Fgf4−/− mouse ESCs fail to induce Gata4-expressing PrE cells in monolayer cultures without LIF^16^. This ability was shown to be rescued by the addition of FGF4, indicating that the autocrine production of Fgf4 is necessary for PrE differentiation of mouse ESCs. Raina et al. examined how the frequency of ERK pulses of each cell depend on FGF4 concentration using Fgf4−/− ESC line harboring sensor for ERK activity^27^. In a serum-free culture condition, they demonstrated that the addition of 2.5–20 ng/ml FGF4 induced ERK pulses in Fgf4−/− cells which showed almost no pulse without FGF4, and the frequency of ERK pulses was increased in a dose-dependent manner of FGF4^27^. They also showed that the proportion of cells that differentiated into the PrE cell lineage was increased in a dose-dependent manner of FGF4 in the same condition^34^. In our system, ERK pulse activity of cells under -LIF condition also may be affected by the addition of FGF4, but signals from endogenous Fgf4 and possibly serum components may have covered the effects of additional FGF4 for frequency of ERK pulses and efficiency of PrE differentiation. It may be for the same reason why active/inactive status did not seem to be affected by addition of an excess amount of FGF4 in our system. Since LIF is known to activate not only JAK/STAT pathway, but also ERK pathway in ESCs^35^, it may be possible that presence of LIF slightly enhance activation of ERK by FGF4 in our system, and an increase of pulse frequency could be barely detected only in + LIF condition.

Finally, we found that a significantly wider pulse (18–20 min) was observed in all cells after addition of a Raf inhibitor (Fig. 4C, S7E, S8, S9, and supplementary movie 4). The width of the ERK pulse was drastically affected, and the long pulse (probably aggregation of pulses) was detected in all observed cells (N = 46, Fig. 4C, S7E, and S8). This result indicates that Raf activity plays a critical role in modulation of frequency of ERK pulses. The widened, synchronous pulse induced by the Raf inhibitor was observed in all cells in the colony, even in inactive cells (Fig. 4C and D). Since Raf inhibitors are known to cause Raf activation (paradoxical activation^25^), the wide pulse observed in our experiment is assumed to be the result of Raf activation. Given that, forced activation of Raf could overcome inactive state and generate an ERK pulse even in inactive cells, suggesting that Raf may be located downstream of factors that suppresses the generation of ERK pulse in inactive cells, and Raf activity may be regulated by such a factors. It was shown that Raf activation by inhibitor also widened the ERK pulses in somatic cell lines^19^, in which the ERK pulse frequency controlled the cell proliferation rate while ERK signaling is responsible for promotion of cell differentiation in ES cells. Based on these observations, Raf activity is assumed to play a key role in generating appropriate patterns of ERK pulses in ESCs as shown in several somatic cells, and is essential for maintaining the differentiation potential of ESCs.

Taken together, our observations and those of previous studies demonstrated that 1) ERK activity is pulsatile in mouse ESCs, 2) ERK-active and -inactive subpopulations exist in ESC culture, 3) Addition of FGF4 can alter ERK activity in the serum-free, intrinsic Fgf4-free culture as Raina et al. demonstrated, but do not effectively work in serum-containing culture of WT ESCs, and 4) Raf activity regulates the generation of appropriate pattern of ERK pulses, and is probably related to the decision of the active/inactive state.

The exact nature of the correlation between the frequency of ERK pulses in ESCs and PrE/EPI fate determination remains unclear, as does the mechanism of the conversion between active and inactive states. To answer these issues, it is necessary to develop a live imaging system by which we can trace the final cell fates of active and inactive cells.

## Materials and methods

### ESC culture and introduction of FRET biosensor

EB5 feeder-free ESCs, a subline of E14TG2a ESC in which one of Oct4 loci is inserted with blasticidin resistant gene were cultured in DMEM medium (Sigma) supplemented with 10% fetal calf serum (FCS), 1 mM sodium pyruvate, 10–4 M 2-mercaptoethanol, 1 × nonessential amino acids and 1000 U LIF per ml on gelatin-coated dishes. To establish EKAREV-NLS-EB5, a piggy-BAC vector for FRET biosensor EKAREV-NLS and pCAG-PBase transposase expression vector were introduced to EB5 by lipofection using Lipofectamine 2000 (Thermo Fisher Scientific) and selected by puromycin (1 ug/mL). To exclude differentiating (Oct4 negative) cells, 10 μg/ml of blasticidin S was added to the medium when EKAREV-NLS-EB5 were cultured in maintenance condition. Gata6-EGFP ESCs were established from a blastocyst of a mouse line generated by injection of BAC into fertilized eggs. BAC containing flanking region of mouse Gata6 locus was purchased from BACPAC resources and modified by BAC modification system developed by Copeland et al.^36^ to be inserted with EGFP into the start codon of Gata6 to report expression of endogenous Gata6 by EGFP fluorescence. A BAC-Tg mouse line was selected by checking EGFP fluorescence in preimplantation embryos was consistent with intrinsic Gata6 protein distribution detected by immunofluorescence (Fig. S6B). ESCs were established using a modified version of a previous protocol^37–39^. Briefly, blastocysts at E3.5 were collected from BAC-Tg mice, and cultured in ESGRO complete basal medium (Millipore) supplemented with 0.4 μM PD0325901 (Cayman Chemical), 3 μM CHIR99021 (Wako) and 1000 units/ml leukocyte inhibitory factor (LIF, Millipore), on mouse embryonic fibroblast (MEF) feeder cells (ReproCELL). After 4–7 days, we trypsinized the clump, replated into new wells, and expanded enough to be served for experiment. Differentiation ability of reporter ESCs into PrE cells was examined by embryoid body formation (Fig. S6E).

### Live imaging of EKAREV-NLS-EB5 ESCs and processing of FRET/CFP data

EKAREV-NLS-EB5 ESCs were seeded on gelatin-coated glass-bottom dishes (Mat-Teck) and incubated in an air-conditioned chamber maintained at 37 °C during live imaging. Since one of Oct4 loci of EB5 is inserted with blasticidin resistant gene, 10 μg/ml blasticidin S was continuously added when observation was performed in maintenance condition. Images of CFP and YFP fluorescence were acquired using a confocal laser scanning microscope system (Nikon A1, Nikon) under the condition for only CFP was excited by 405 nm blue laser. The interval of time frames was 3 min for all experiments. Raw images were processed to make FRET ratio images, and FRET ratio of individual cell was measured with ROI set in the nucleus of each cell using Metamorph software (Molecular Devices). Cells proliferated during live imaging were excluded from analysis because CFP and YFP fluorescence in proliferating cells were significantly decreased and thought not to show accurate ERK activity. Processing of detrending, detection of peaks, and other statistical analysis of FRET data were performed by Python and R. ERK activation pulses were detected by “detect_peaks.py” (https://github.com/demotu/BMC) with the threshold peak value (mph) is 1.2 and minimum peak distance (mpd) is 3. The cross-correlation was calculated by python with “numpy.correlate” function. We defined cells exhibiting one or more pulses during live imaging for more than 120 min as active cells, and those showing no obvious pulses throughout live imaging as inactive cells. Only cells that successfully measured data for more than 160 min exhibiting no pulse were classified as inactive cells.

### FACS analysis

FACS analysis of Gata6-EGFP ESCs was performed using a cell sorter SH800S (SONY). Intensity of EGFP fluorescence was measured as an indication of differentiation of Gata6 + PrE cells. Propidium Iodide (PI) was used for counterstain to exclude dead cells from analysis.

### Immunocytochemistry and immunohistochemistry

EB5 ESCs on plates were fixed with 4% PFA/PBS for about 16 h at 4 °C, washed three times with PBS, incubated with 0.3% Triton-X100 for 30 min at room temperature (RT), and blocked with BSA/PBS for 1 h at RT. Following incubation with goat anti-Gata4 (Santa Cruz) overnight at 4 °C, the cells were washed and incubated with secondary Alexa Fluor-conjugated antibodies (Thermo Fisher Scientific) at a dilution of 1:1000 for 30 min at RT. Hoechst 33,342 (Thermo Fisher Scientific) was used to counterstain of the nuclei. Observations and acquisition of images were performed with an inverted microscope system (IX-70, Olympus). Blastocysts collected from Gata6-EGFP reporter mouse line were fixed with 4% PFA/PBS for about 16 h at 4 °C, washed with PBS, incubated with 0.3% Triton-X100 for 30 min at room temperature (RT), and blocked with 2% BSA/PBS for 1 h at RT. Blastocysts were incubated with mouse monoclonal anti-GFP (Abcam) goat polyclonal and/or anti-Gata6 (R&D Systems) overnight at 4 °C, then washed and incubated with secondary Alexa Fluor-conjugated antibodies (Thermo Fisher Scientific) at a dilution of 1:1000 for 30 min at RT. Images of dissected 7.5 dpc embryos and embryoid bodies generated from Gata6-EGFP ESCs were acquired without fixation. Adult intestine of Gata6-EGFP reporter mouse line were dissected and fixed with 4% PFA/PBS for about 16 h at 4 °C, and processed into frozen sections. Observations and acquisition of images were performed with Nikon A1 system. Hoechst 33,342 was used to counterstain of the nuclei.

## Supplementary Information

Supplementary Figures.

Supplementary Legends.

Supplementary Moive S1.

Supplementary Moive S2.

Supplementary Moive S3.

Supplementary Moive S4.

## Data Availability

The datasets used and/or analyzed during the current study available from the corresponding author on reasonable request.
