# Supplementary material for: Generation of pulsatile ERK activity in mouse embryonic stem cells is regulated by Raf activity
Source: Sci Rep. 2023 Jun 10;13:9465. doi: 10.1038/s41598-023-36424-6 (PMC10257726; doi:10.1038/s41598-023-36424-6)
Supplement: Supplementary file 1 — Supplementary Figures. [file 41598_2023_36424_MOESM1_ESM.pptx]

## Slide 1
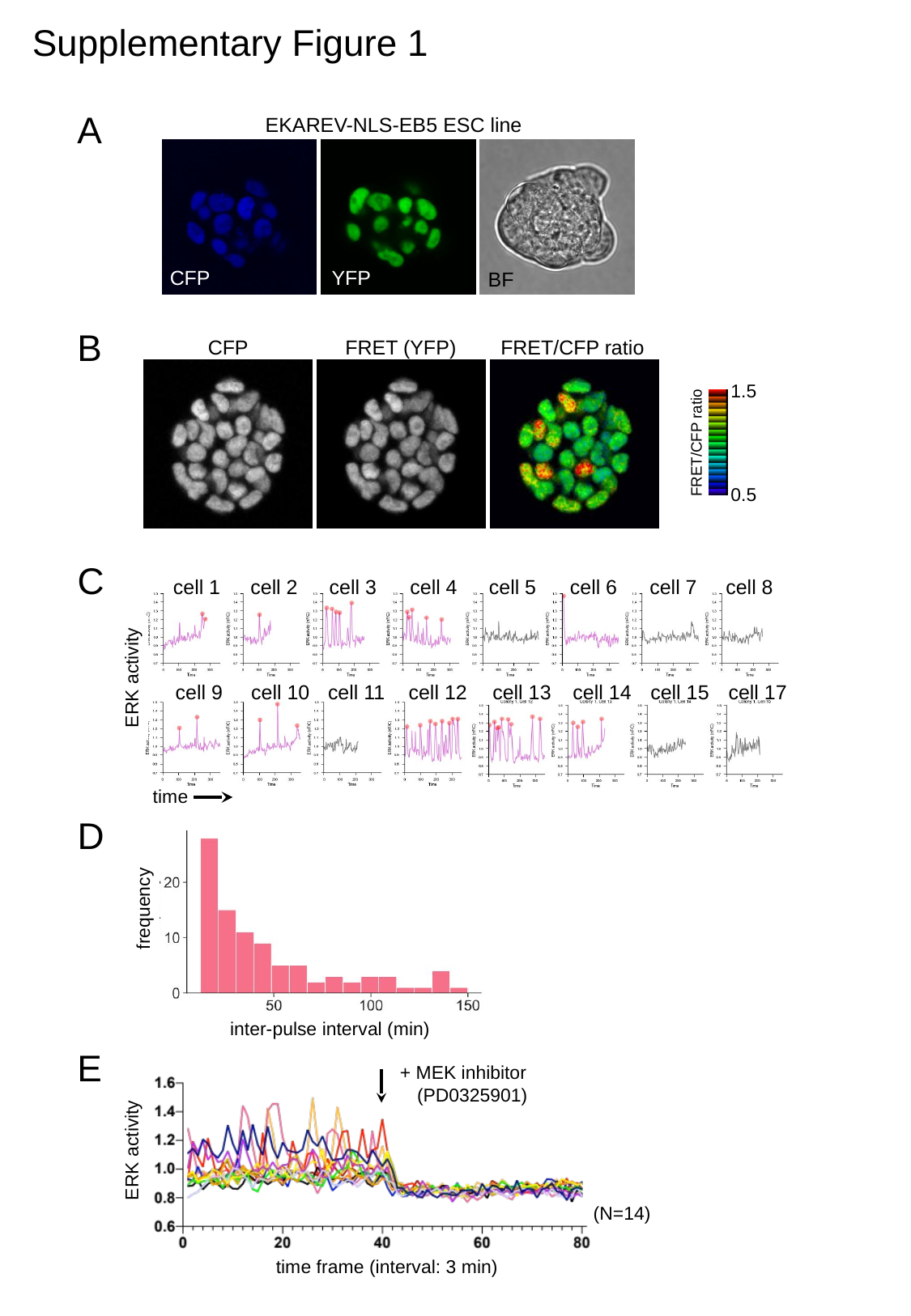

Supplementary Figure 1
A
EKAREV-NLS-EB5 ESC line
CFP
YFP
BF
B
CFP
FRET (YFP)
FRET/CFP ratio
1.5
FRET/CFP ratio
0.5
C
cell 1
cell 2
cell 3
cell 4
cell 5
cell 6
cell 7
cell 8
ERK activity
cell 9
cell 10
cell 11
cell 12
cell 13
cell 14
cell 15
cell 17
time
D
frequency
inter-pulse interval (min)
E
+ MEK inhibitor
(PD0325901)
ERK activity
(N=14)
time frame (interval: 3 min)

## Slide 2
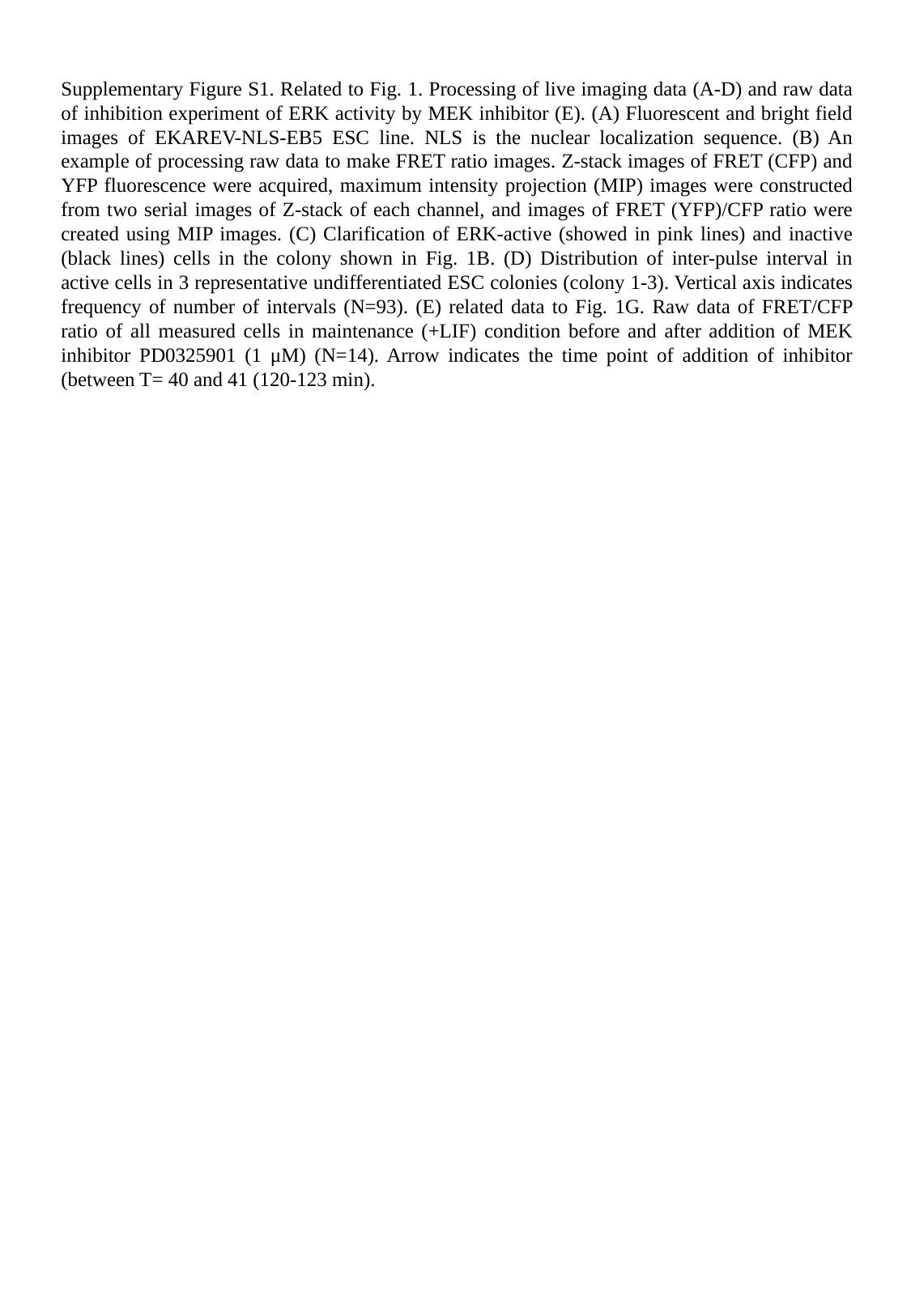

Supplementary Figure S1. Related to Fig. 1. Processing of live imaging data (A-D) and raw data of inhibition experiment of ERK activity by MEK inhibitor (E). (A) Fluorescent and bright field images of EKAREV-NLS-EB5 ESC line. NLS is the nuclear localization sequence. (B) An example of processing raw data to make FRET ratio images. Z-stack images of FRET (CFP) and YFP fluorescence were acquired, maximum intensity projection (MIP) images were constructed from two serial images of Z-stack of each channel, and images of FRET (YFP)/CFP ratio were created using MIP images. (C) Clarification of ERK-active (showed in pink lines) and inactive (black lines) cells in the colony shown in Fig. 1B. (D) Distribution of inter-pulse interval in active cells in 3 representative undifferentiated ESC colonies (colony 1-3). Vertical axis indicates frequency of number of intervals (N=93). (E) related data to Fig. 1G. Raw data of FRET/CFP ratio of all measured cells in maintenance (+LIF) condition before and after addition of MEK inhibitor PD0325901 (1 μM) (N=14). Arrow indicates the time point of addition of inhibitor (between T= 40 and 41 (120-123 min).

## Slide 3
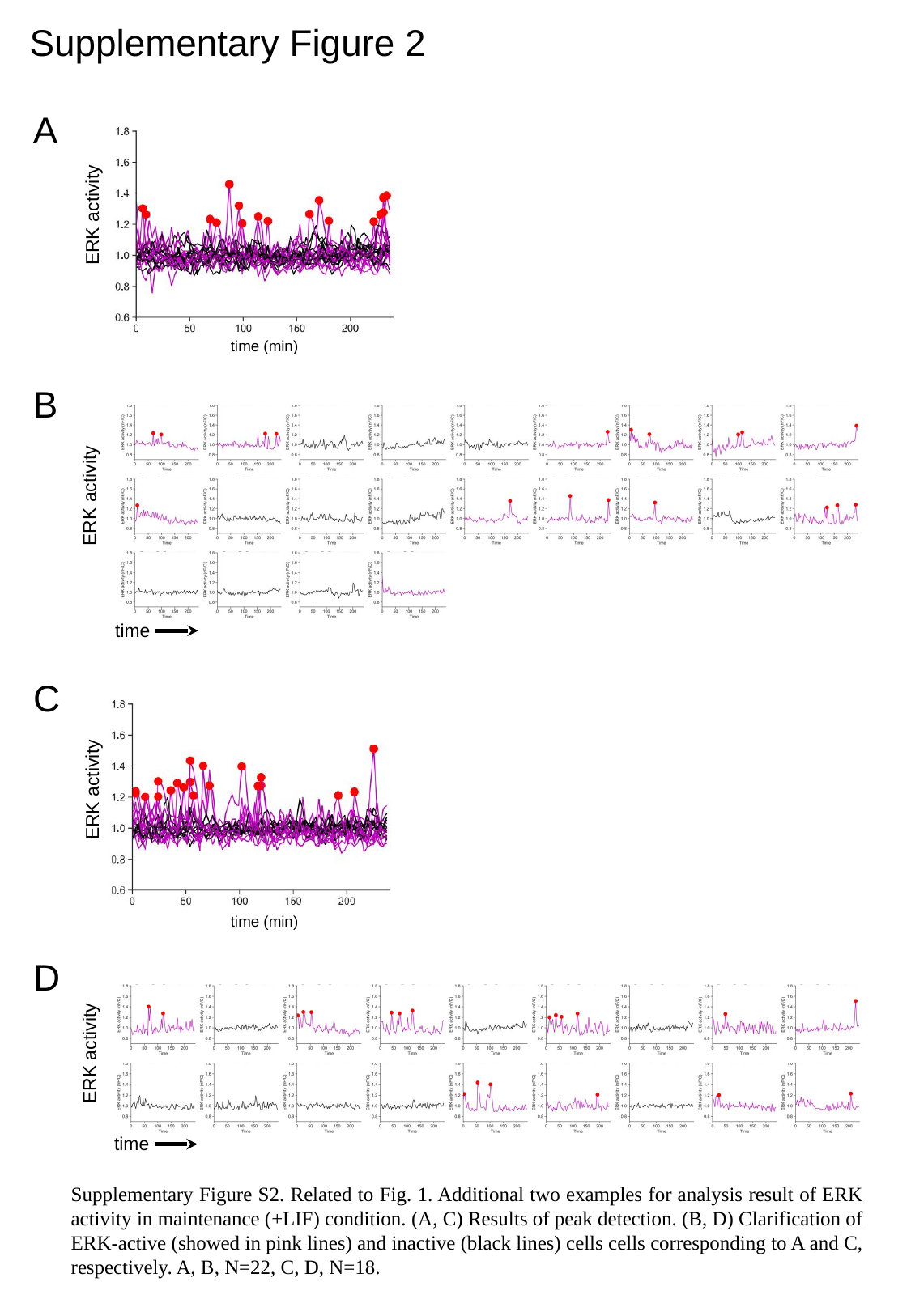

Supplementary Figure 2
A
ERK activity
time (min)
B
ERK activity
time
C
ERK activity
time (min)
D
ERK activity
time
Supplementary Figure S2. Related to Fig. 1. Additional two examples for analysis result of ERK activity in maintenance (+LIF) condition. (A, C) Results of peak detection. (B, D) Clarification of ERK-active (showed in pink lines) and inactive (black lines) cells cells corresponding to A and C, respectively. A, B, N=22, C, D, N=18.

## Slide 4
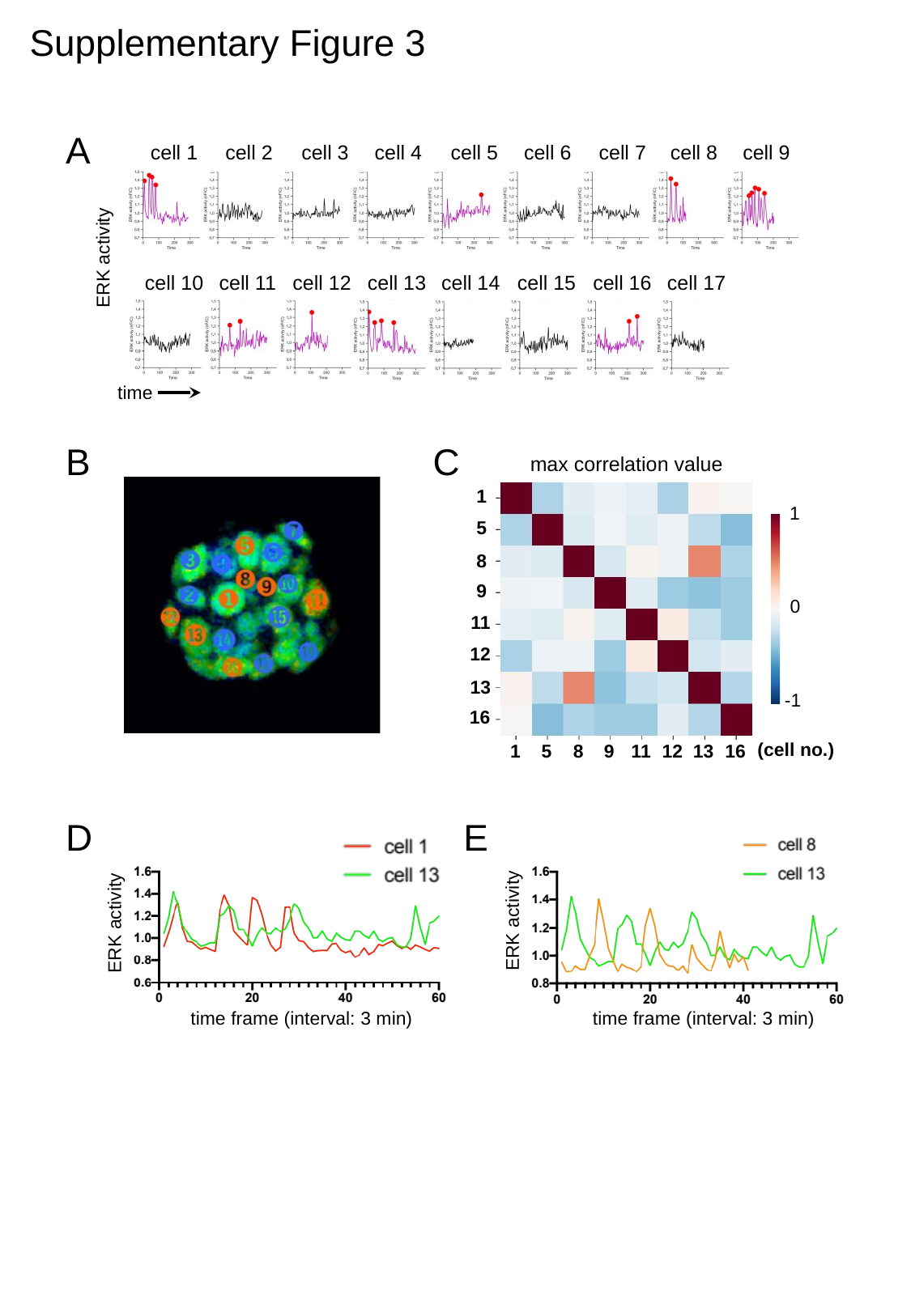

Supplementary Figure 3
A
cell 1
cell 2
cell 3
cell 4
cell 5
cell 6
cell 7
cell 8
cell 9
ERK activity
cell 10
cell 11
cell 12
cell 13
cell 14
cell 15
cell 16
cell 17
time
B
C
max correlation value
1
1
5
8
9
0
11
12
13
-1
16
1
5
8
9
11
12
13
16
(cell no.)
D
E
ERK activity
ERK activity
time frame (interval: 3 min)
time frame (interval: 3 min)

## Slide 5
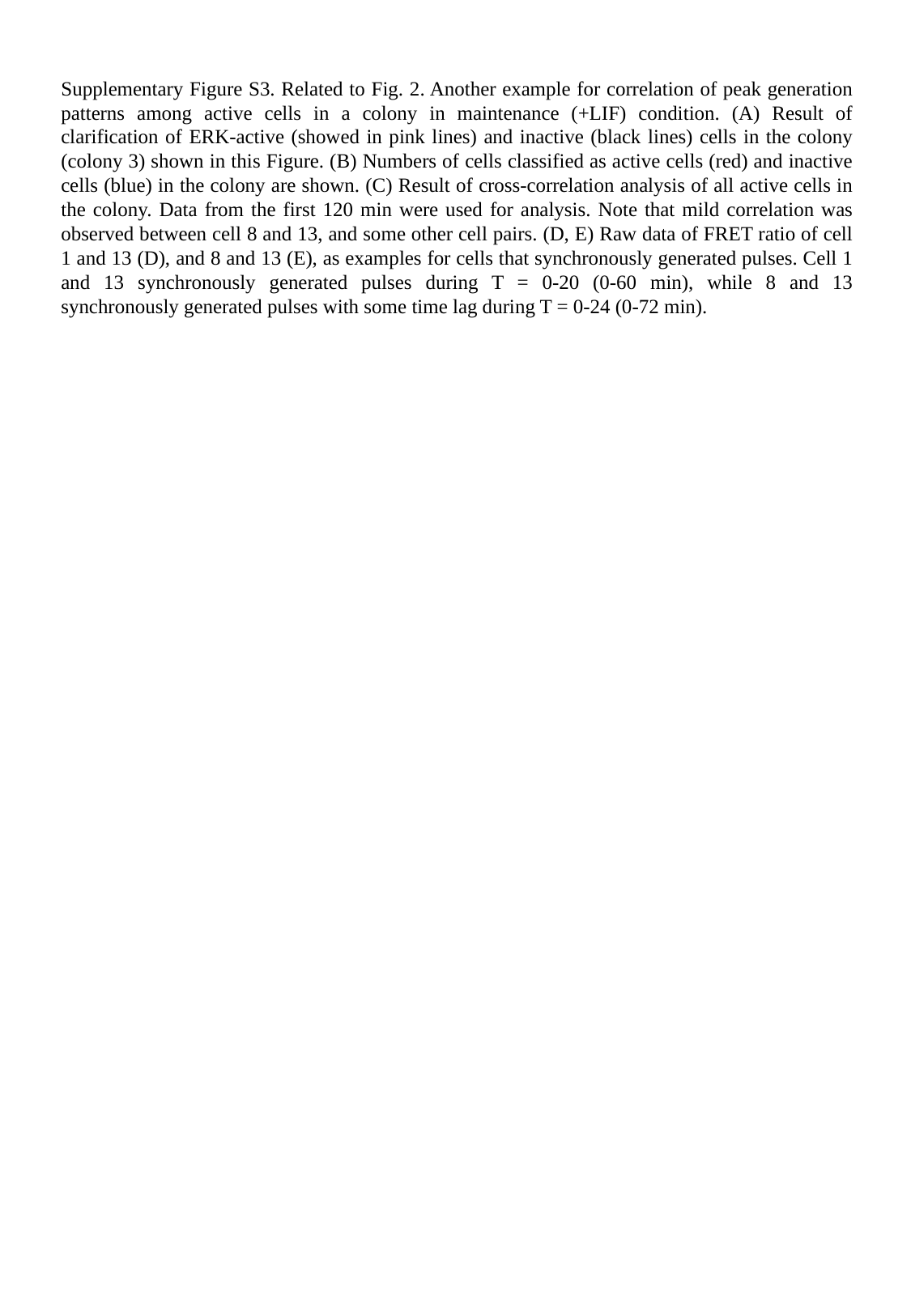

Supplementary Figure S3. Related to Fig. 2. Another example for correlation of peak generation patterns among active cells in a colony in maintenance (+LIF) condition. (A) Result of clarification of ERK-active (showed in pink lines) and inactive (black lines) cells in the colony (colony 3) shown in this Figure. (B) Numbers of cells classified as active cells (red) and inactive cells (blue) in the colony are shown. (C) Result of cross-correlation analysis of all active cells in the colony. Data from the first 120 min were used for analysis. Note that mild correlation was observed between cell 8 and 13, and some other cell pairs. (D, E) Raw data of FRET ratio of cell 1 and 13 (D), and 8 and 13 (E), as examples for cells that synchronously generated pulses. Cell 1 and 13 synchronously generated pulses during T = 0-20 (0-60 min), while 8 and 13 synchronously generated pulses with some time lag during T = 0-24 (0-72 min).

## Slide 6
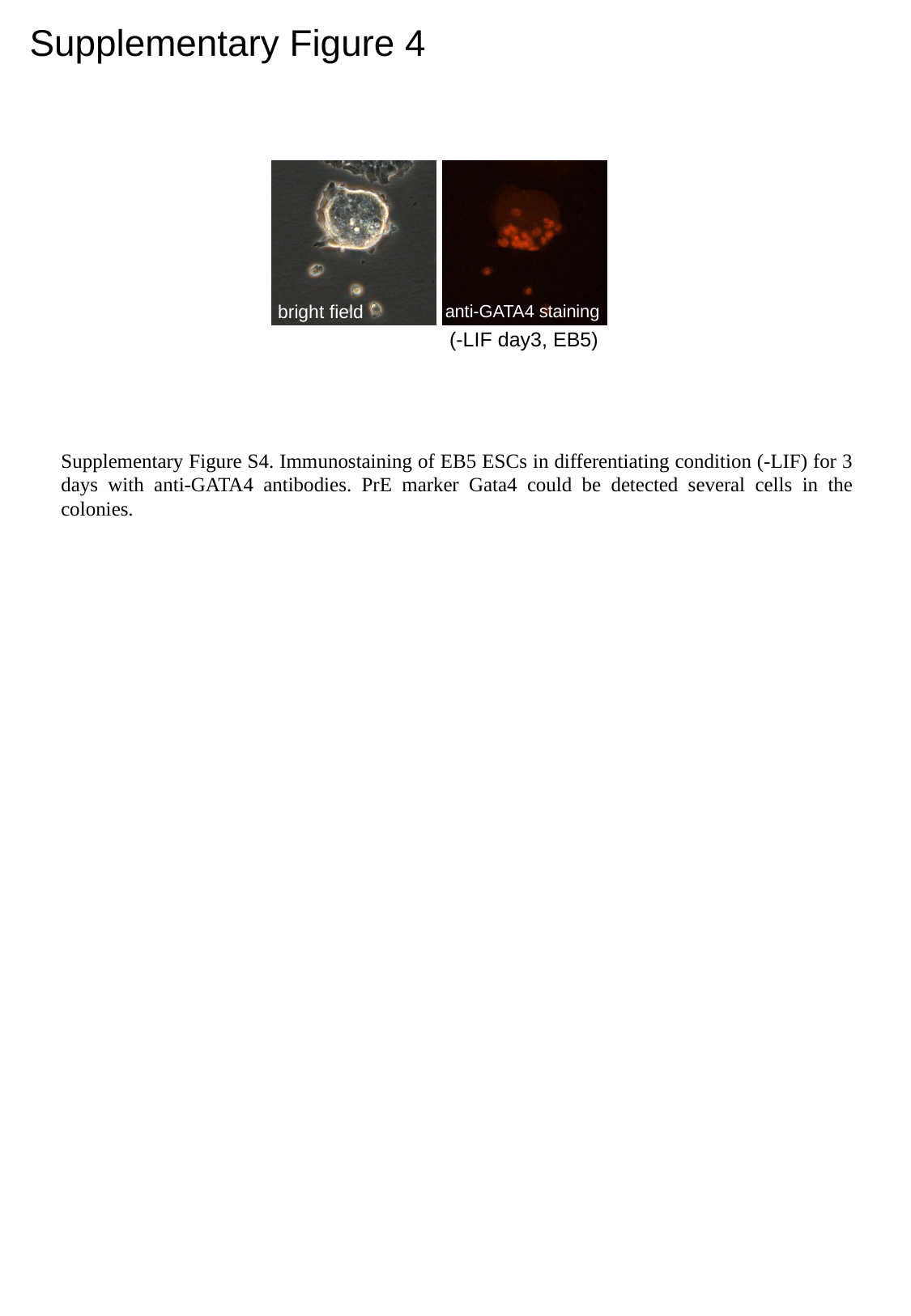

Supplementary Figure 4
bright field
anti-GATA4 staining
(-LIF day3, EB5)
Supplementary Figure S4. Immunostaining of EB5 ESCs in differentiating condition (-LIF) for 3 days with anti-GATA4 antibodies. PrE marker Gata4 could be detected several cells in the colonies.

## Slide 7
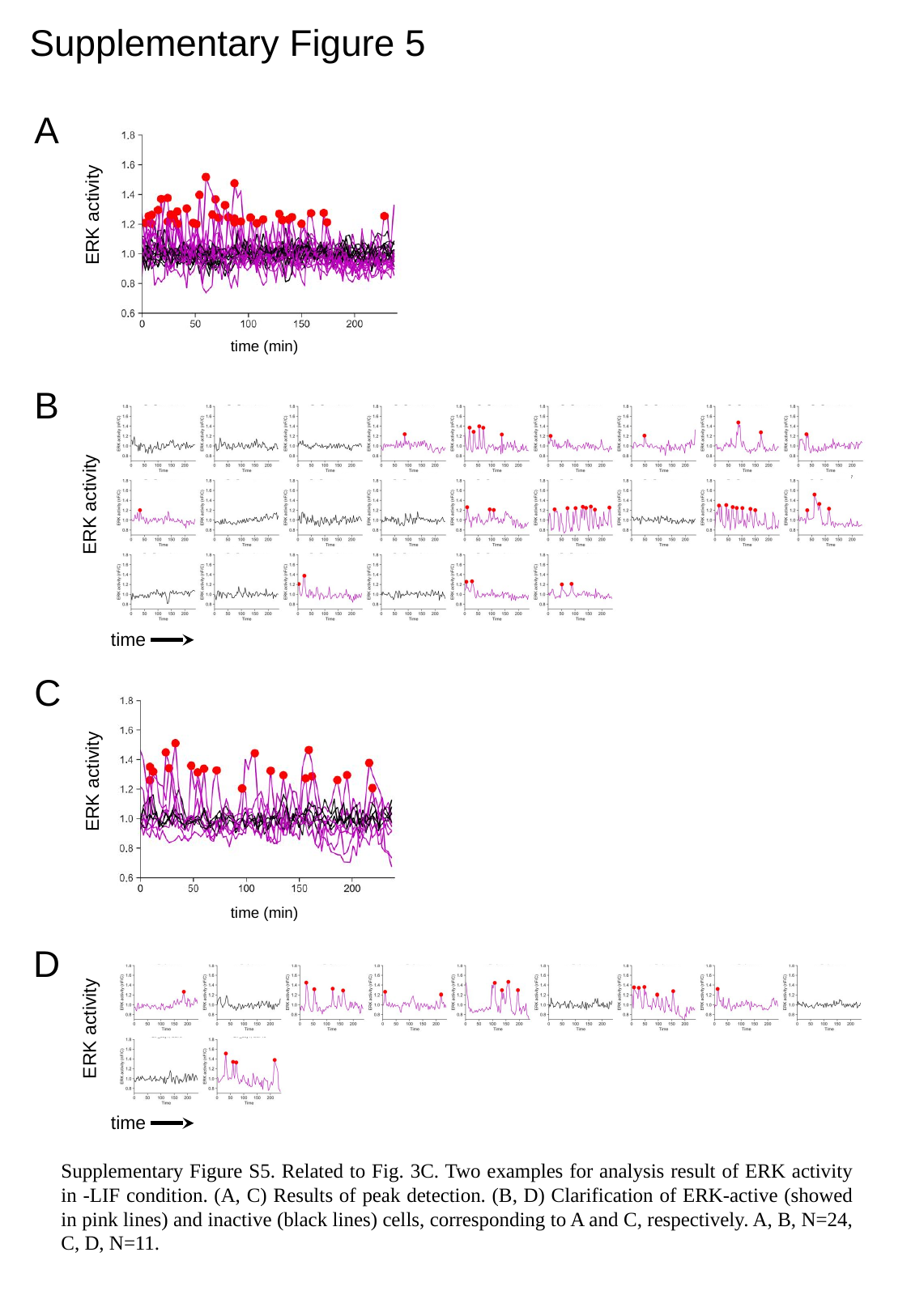

Supplementary Figure 5
A
ERK activity
time (min)
B
ERK activity
time
C
ERK activity
time (min)
D
ERK activity
time
Supplementary Figure S5. Related to Fig. 3C. Two examples for analysis result of ERK activity in -LIF condition. (A, C) Results of peak detection. (B, D) Clarification of ERK-active (showed in pink lines) and inactive (black lines) cells, corresponding to A and C, respectively. A, B, N=24, C, D, N=11.

## Slide 8
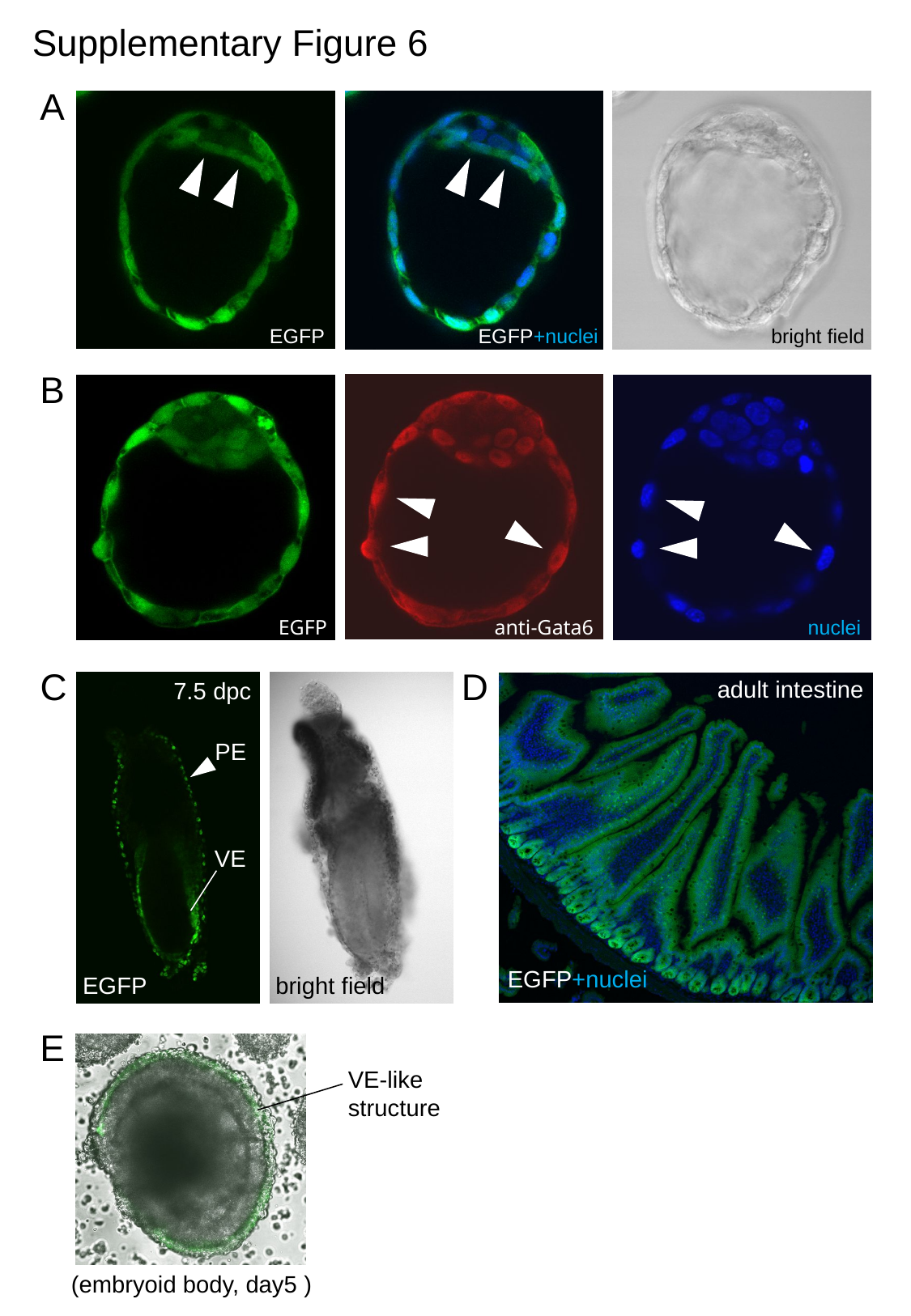

Supplementary Figure 6
A
EGFP
EGFP+nuclei
bright field
B
EGFP
anti-Gata6
nuclei
C
D
adult intestine
7.5 dpc
PE
VE
EGFP+nuclei
EGFP
bright field
E
VE-like structure
(embryoid body, day5 )

## Slide 9
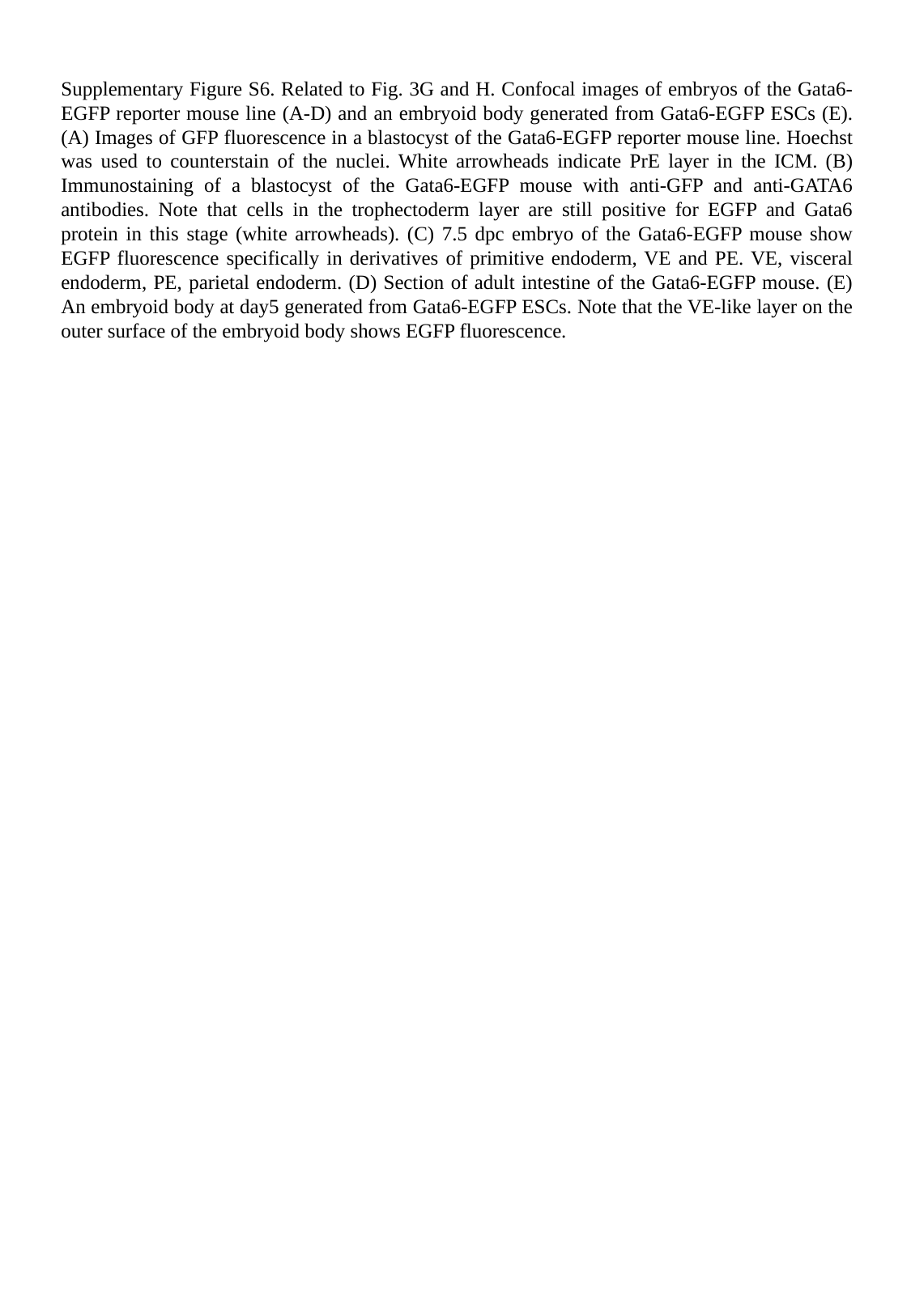

Supplementary Figure S6. Related to Fig. 3G and H. Confocal images of embryos of the Gata6-EGFP reporter mouse line (A-D) and an embryoid body generated from Gata6-EGFP ESCs (E). (A) Images of GFP fluorescence in a blastocyst of the Gata6-EGFP reporter mouse line. Hoechst was used to counterstain of the nuclei. White arrowheads indicate PrE layer in the ICM. (B) Immunostaining of a blastocyst of the Gata6-EGFP mouse with anti-GFP and anti-GATA6 antibodies. Note that cells in the trophectoderm layer are still positive for EGFP and Gata6 protein in this stage (white arrowheads). (C) 7.5 dpc embryo of the Gata6-EGFP mouse show EGFP fluorescence specifically in derivatives of primitive endoderm, VE and PE. VE, visceral endoderm, PE, parietal endoderm. (D) Section of adult intestine of the Gata6-EGFP mouse. (E) An embryoid body at day5 generated from Gata6-EGFP ESCs. Note that the VE-like layer on the outer surface of the embryoid body shows EGFP fluorescence.

## Slide 10
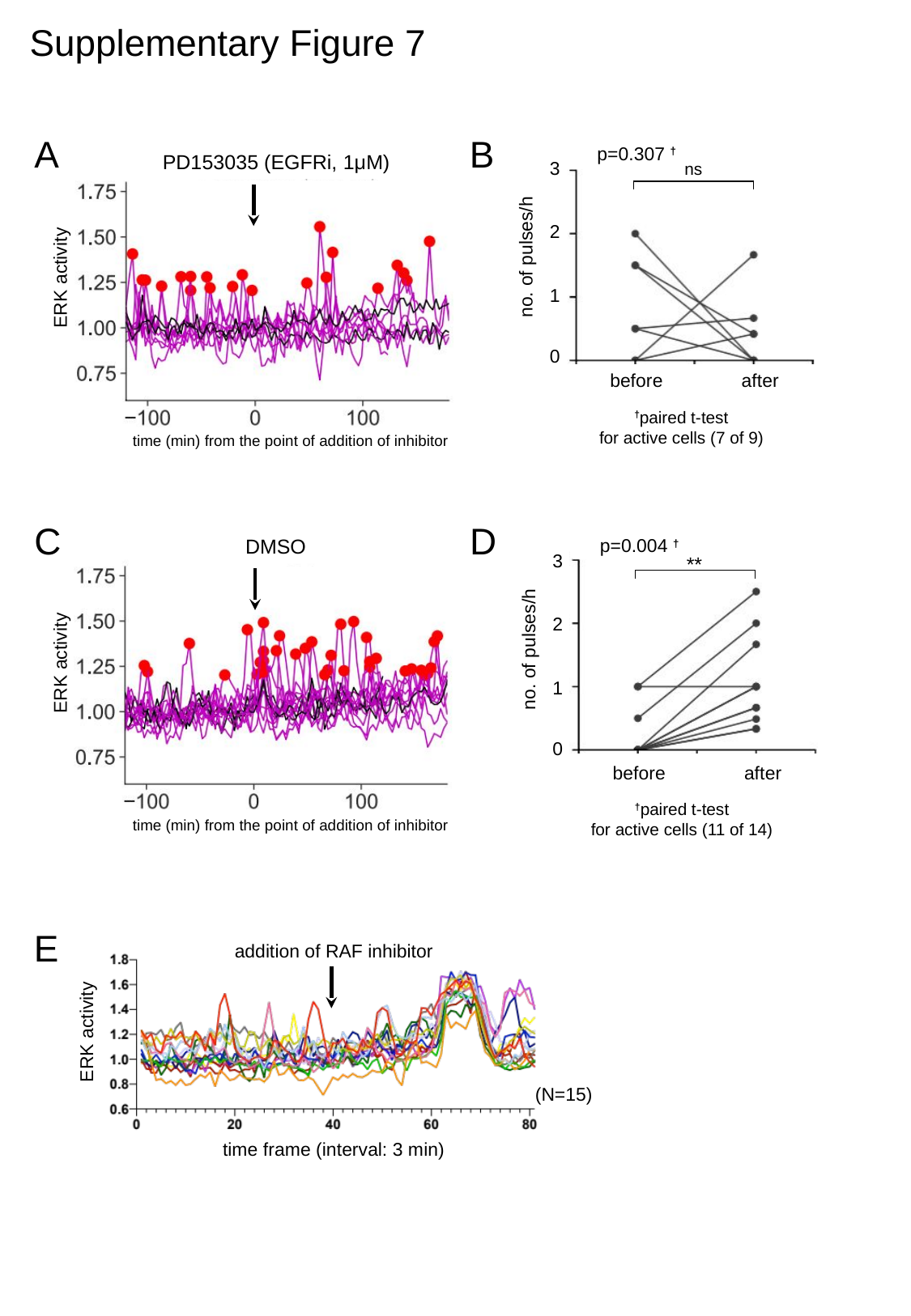

Supplementary Figure 7
p=0.307 †
3
ns
2
no. of pulses/h
1
0
before after
†paired t-test
for active cells (7 of 9)
A
B
PD153035 (EGFRi, 1μM)
ERK activity
time (min) from the point of addition of inhibitor
C
D
p=0.004 †
3
**
2
no. of pulses/h
1
0
before after
†paired t-test
for active cells (11 of 14)
DMSO
ERK activity
time (min) from the point of addition of inhibitor
E
addition of RAF inhibitor
ERK activity
(N=15)
time frame (interval: 3 min)

## Slide 11
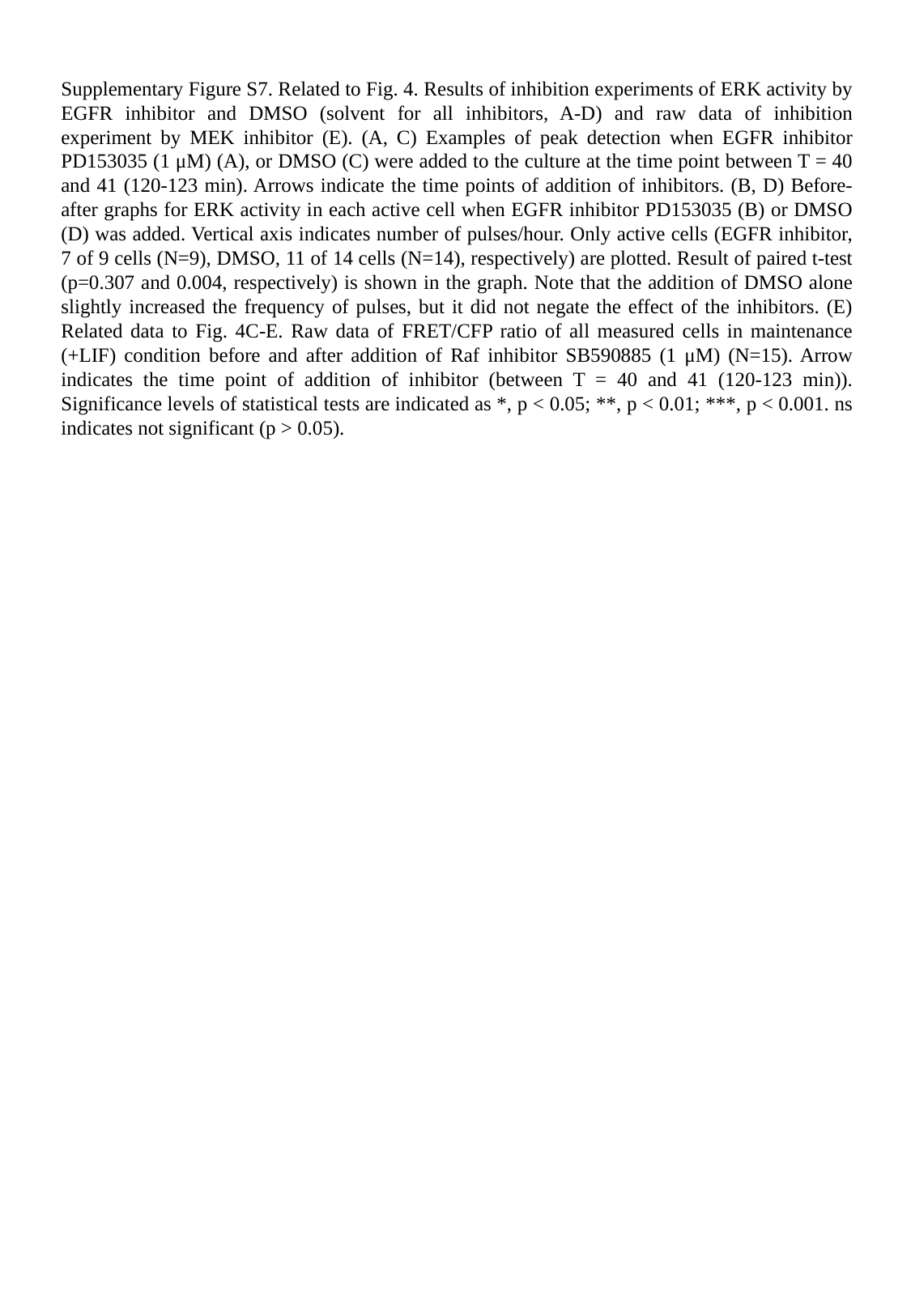

Supplementary Figure S7. Related to Fig. 4. Results of inhibition experiments of ERK activity by EGFR inhibitor and DMSO (solvent for all inhibitors, A-D) and raw data of inhibition experiment by MEK inhibitor (E). (A, C) Examples of peak detection when EGFR inhibitor PD153035 (1 μM) (A), or DMSO (C) were added to the culture at the time point between T = 40 and 41 (120-123 min). Arrows indicate the time points of addition of inhibitors. (B, D) Before-after graphs for ERK activity in each active cell when EGFR inhibitor PD153035 (B) or DMSO (D) was added. Vertical axis indicates number of pulses/hour. Only active cells (EGFR inhibitor, 7 of 9 cells (N=9), DMSO, 11 of 14 cells (N=14), respectively) are plotted. Result of paired t-test (p=0.307 and 0.004, respectively) is shown in the graph. Note that the addition of DMSO alone slightly increased the frequency of pulses, but it did not negate the effect of the inhibitors. (E) Related data to Fig. 4C-E. Raw data of FRET/CFP ratio of all measured cells in maintenance (+LIF) condition before and after addition of Raf inhibitor SB590885 (1 μM) (N=15). Arrow indicates the time point of addition of inhibitor (between T = 40 and 41 (120-123 min)). Significance levels of statistical tests are indicated as *, p < 0.05; **, p < 0.01; ***, p < 0.001. ns indicates not significant (p > 0.05).

## Slide 12
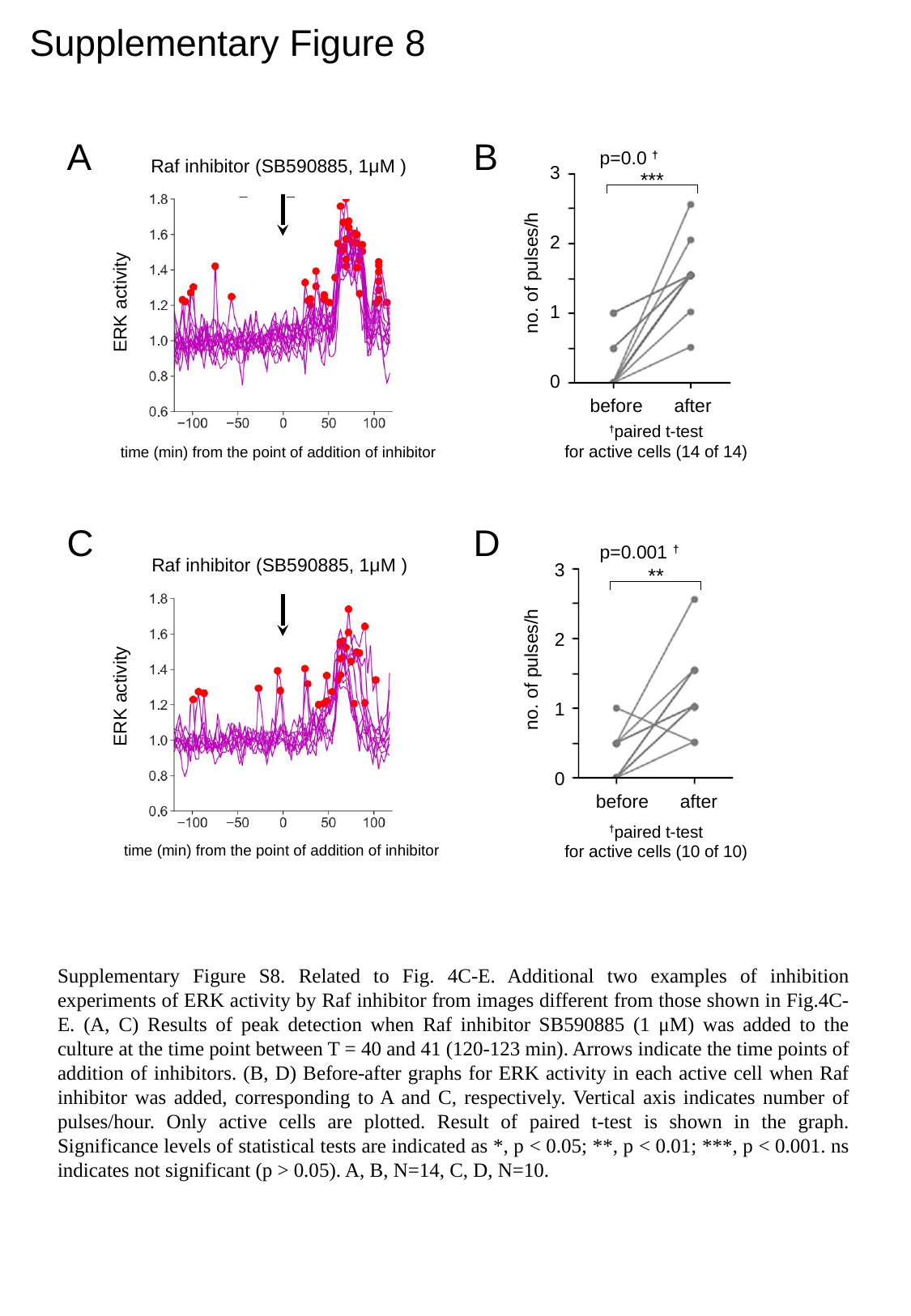

Supplementary Figure 8
A
B
p=0.0 †
Raf inhibitor (SB590885, 1μM )
3
***
2
no. of pulses/h
ERK activity
1
0
before after
†paired t-test
for active cells (14 of 14)
time (min) from the point of addition of inhibitor
C
D
p=0.001 †
Raf inhibitor (SB590885, 1μM )
3
**
2
no. of pulses/h
ERK activity
1
0
before after
†paired t-test
for active cells (10 of 10)
time (min) from the point of addition of inhibitor
Supplementary Figure S8. Related to Fig. 4C-E. Additional two examples of inhibition experiments of ERK activity by Raf inhibitor from images different from those shown in Fig.4C-E. (A, C) Results of peak detection when Raf inhibitor SB590885 (1 μM) was added to the culture at the time point between T = 40 and 41 (120-123 min). Arrows indicate the time points of addition of inhibitors. (B, D) Before-after graphs for ERK activity in each active cell when Raf inhibitor was added, corresponding to A and C, respectively. Vertical axis indicates number of pulses/hour. Only active cells are plotted. Result of paired t-test is shown in the graph. Significance levels of statistical tests are indicated as *, p < 0.05; **, p < 0.01; ***, p < 0.001. ns indicates not significant (p > 0.05). A, B, N=14, C, D, N=10.

## Slide 13
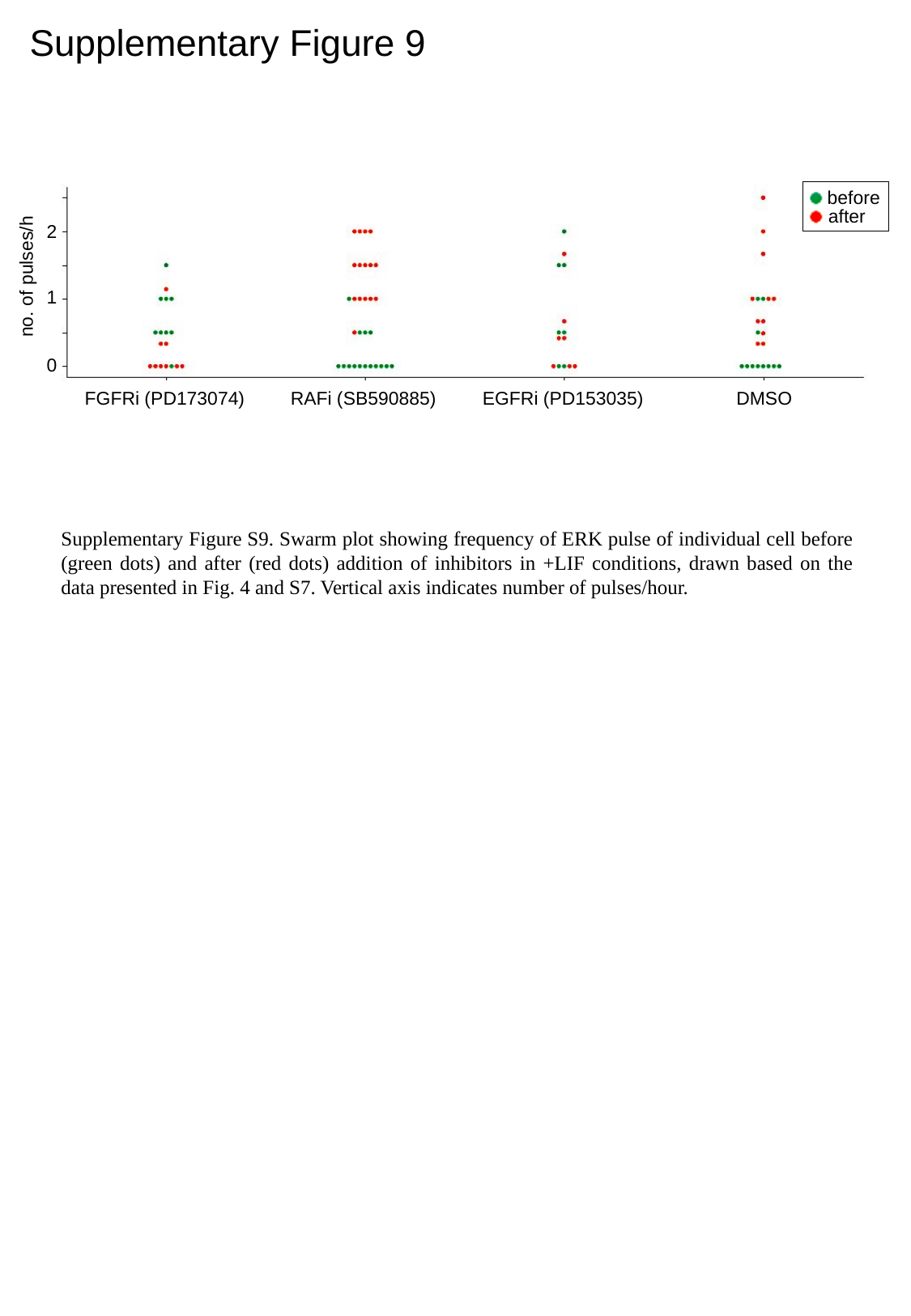

Supplementary Figure 9
before
after
2
no. of pulses/h
1
0
FGFRi (PD173074)
RAFi (SB590885)
EGFRi (PD153035)
DMSO
Supplementary Figure S9. Swarm plot showing frequency of ERK pulse of individual cell before (green dots) and after (red dots) addition of inhibitors in +LIF conditions, drawn based on the data presented in Fig. 4 and S7. Vertical axis indicates number of pulses/hour.
