# Supplementary material for: Generation of pulsatile ERK activity in mouse embryonic stem cells is regulated by Raf activity
Source: Sci Rep. 2023 Jun 10;13:9465. doi: 10.1038/s41598-023-36424-6 (PMC10257726; doi:10.1038/s41598-023-36424-6)
Supplement: Supplementary file 2 — Supplementary Legends. [file 41598_2023_36424_MOESM2_ESM.pptx]

## Slide 1
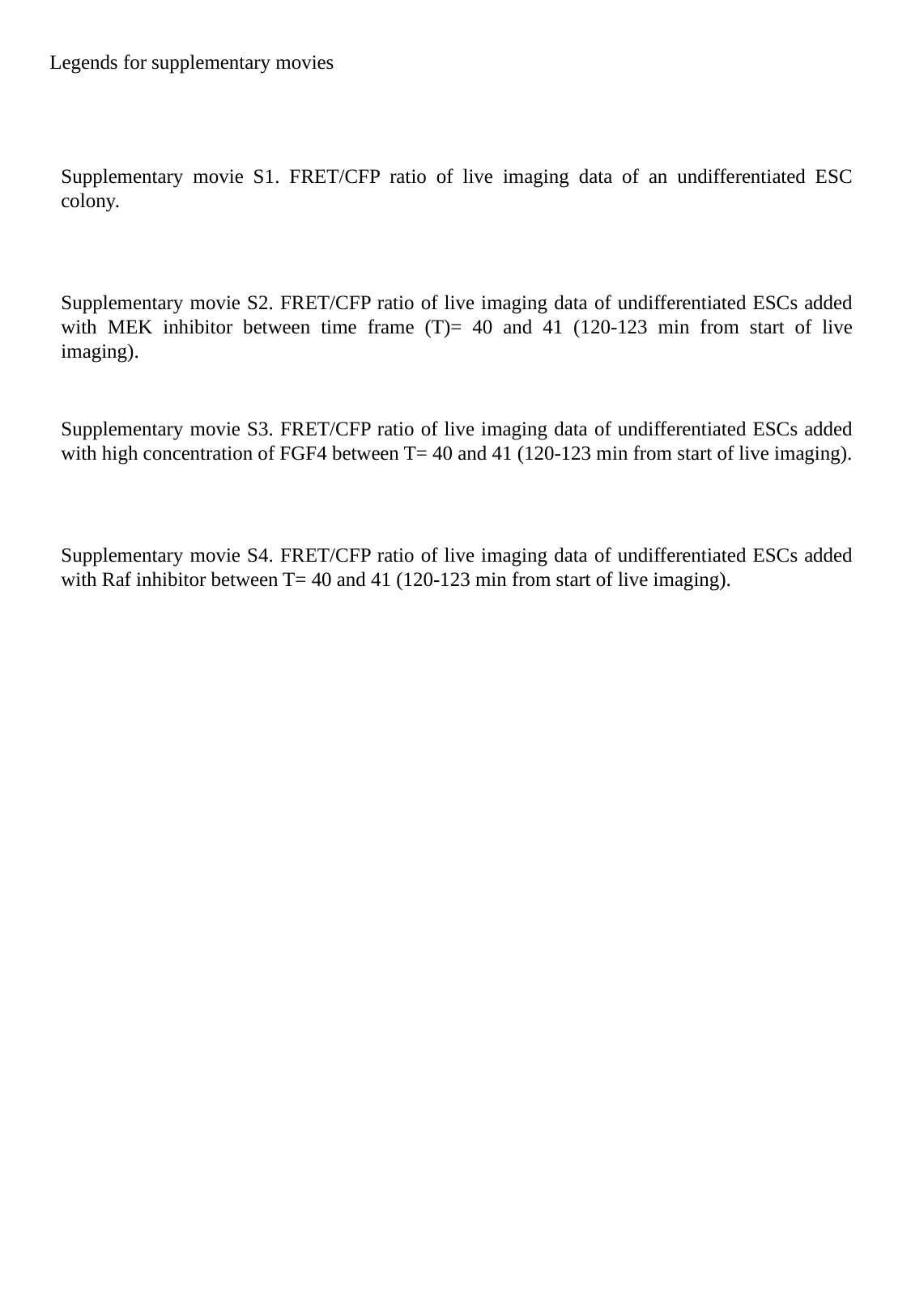

Legends for supplementary movies
Supplementary movie S1. FRET/CFP ratio of live imaging data of an undifferentiated ESC colony.
Supplementary movie S2. FRET/CFP ratio of live imaging data of undifferentiated ESCs added with MEK inhibitor between time frame (T)= 40 and 41 (120-123 min from start of live imaging).
Supplementary movie S3. FRET/CFP ratio of live imaging data of undifferentiated ESCs added with high concentration of FGF4 between T= 40 and 41 (120-123 min from start of live imaging).
Supplementary movie S4. FRET/CFP ratio of live imaging data of undifferentiated ESCs added with Raf inhibitor between T= 40 and 41 (120-123 min from start of live imaging).
